# Supplementary material for: Self-reported knowledge attitude and practice of healthcare professionals in the management of infection and antimicrobial stewardship: a systematic review
Source: JAC Antimicrob Resist. 2025 Sep 16;7(5):dlaf159. doi: 10.1093/jacamr/dlaf159 (PMC12448720; doi:10.1093/jacamr/dlaf159)
Supplement: dlaf159_Supplementary_Data [file dlaf159_supplementary_data.docx]

**Supplementary file**

**Section 1: Medline search strategy**

Searches run: 24 Jan 2024

Ovid MEDLINE(R) ALL <1946 to January 23, 2024>

1 Drug Resistance, Microbial/ 61621

2 exp Drug Resistance, Bacterial/ 103770

3 Antimicrobial Stewardship/ 3504

4 antimicrobial resistan*.mp. 42262

5 (antimicrobial adj3 ("use" or stewardship or resistan*)).mp. [mp=title, book title, abstract, original title, name of substance word, subject heading word, floating sub-heading word, keyword heading word, organism supplementary concept word, protocol supplementary concept word, rare disease supplementary concept word, unique identifier, synonyms, population supplementary concept word, anatomy supplementary concept word] 57771

6 Infection Control/ or infection prevention control.mp. 29067

7 exp Anti-Bacterial Agents/tu [Therapeutic Use] 285870

8 exp Antifungal Agents/tu [Therapeutic Use] 55873

9 exp Antiviral Agents/tu [Therapeutic Use] 138482

10 exp Antiparasitic Agents/tu [Therapeutic Use] 89421

11 1 or 2 or 3 or 4 or 5 or 6 or 7 or 8 or 9 or 10 686376

12 exp "Attitude of Health Personnel"/ 170731

13 Health Knowledge, Attitudes, Practice/ 127734

14 Behavior/ or Habits/ 36346

15 (attitude* or knowledge or practice* or opinion* or belief* or perspective* or behaviour* or behavior* or "com-b" or values or values or understanding or awareness or culture).mp. [mp=title, book title, abstract, original title, name of substance word, subject heading word, floating sub-heading word, keyword heading word, organism supplementary concept word, protocol supplementary concept word, rare disease supplementary concept word, unique identifier, synonyms, population supplementary concept word, anatomy supplementary concept word] 7645072

16 12 or 13 or 14 or 15 7667761

17 11 and 16 136460

18 exp Health Personnel/ 625213

19 exp Students/ 172645

20 (careworker* or care worker* or care-worker* or healthcare worker* or health care worker* or health-care worker* or social adult care or heathcare support worker* or health care support worker* or health-care support worker*).mp. [mp=title, book title, abstract, original title, name of substance word, subject heading word, floating sub-heading word, keyword heading word, organism supplementary concept word, protocol supplementary concept word, rare disease supplementary concept word, unique identifier, synonyms, population supplementary concept word, anatomy supplementary concept word] 44203

21 18 or 19 or 20 802141

22 11 and 16 and 21 7083

23 exp Africa, Central/ or exp Africa, Southern/ or exp Africa, Northern/ or exp "Africa South of the Sahara"/ or exp Africa, Western/ or exp Africa, Eastern/ or exp Africa/ or exp South Africa/ 332080

24 exp Latin America/ or exp South America/ 211662

25 exp Asia, Northern/ or exp Asia, Eastern/ or exp Asia, Southern/ or exp Asia/ or exp Asia, Southeastern/ or exp Asia, Central/ or exp Asia, Western/ 1055090

26 23 or 24 or 25 1570628

27 22 not 26 5153

28 limit 27 to yr="2016 - 2024" 2310

29 exp animals/ not humans.sh. 5189902

30 28 not 29 2274

31 limit 30 to english language 2207

32 exp "Surveys and Questionnaires"/ 1228636

33 (questionnaire* or survey*).mp. [mp=title, book title, abstract, original title, name of substance word, subject heading word, floating sub-heading word, keyword heading word, organism supplementary concept word, protocol supplementary concept word, rare disease supplementary concept word, unique identifier, synonyms, population supplementary concept word, anatomy supplementary concept word] 1619033

34 (assess* or tool or tools or instrument* or measur* or scale or index).mp. [mp=title, book title, abstract, original title, name of substance word, subject heading word, floating sub-heading word, keyword heading word, organism supplementary concept word, protocol supplementary concept word, rare disease supplementary concept word, unique identifier, synonyms, population supplementary concept word, anatomy supplementary concept word] 9683886

35 32 or 33 or 34 10452063

36 31 and 35 1466

**Section 2: Study characteristics**

Table 1: Characteristics of the included studies

| **Author, Year** | **Country, Setting** | **Categories of participants, Number (n)** | **Description of questionnaire.**  **Domains measured (total number of items used in questionnaire)** |
| --- | --- | --- | --- |
| Abbas *et al*, 2019^1^ | USA, Hospitals | Nurses (n=159) | Bespoke online tool and administered through REDCAP.  Knowledge and attitude (12) |
| Alcala *et al*, 2018^2^ | USA. Healthcare system | Paediatric healthcare providers  (n=42) | Bespoke online tool administered via email.  Knowledge attitude and practice (NR) |
| Ashiru-Oredope *et al*, 2021^3^ | Multiple countries, Multiple settings | Healthcare Workers (n=18265) | The ECDC questionnaire administered online via social media promotion and project members contacts.  Knowledge attitude and practice (43) |
| Ashiru-Oredope *et al*, 2022^4^ | UK, Multiple settings | Multidisciplinary healthcare workers (n=2404) | The ECDC questionnaire administered online via social media promotion and project members contacts.  Knowledge attitude and practice (43) |
| Barchitta *et al*., 2021^5^ | Italy, NR | Healthcare workers (n=1693) | The ECDC questionnaire administered online.  Knowledge attitude and practice (43) |
| Baudet *et al*., 2020^6^ | France, NR | Postgraduate Dentists (n=455) | Bespoke online questionnaire tool administered via email and social networks.  Knowledge attitude and practice (NR) |
| Belan *et al*., 2021^7^ | France, Nursing home | Medical and nurse coordinators (n=75) | Bespoke tool administered via email (through Survey Monkey) or postal mail.  Attitude and practice (35) |
| Benedict Kpozehouen *et al*., 2023^8^ | Australia, Hospitals | HCWs (n= 43) | Self-administered questionnaire administered during face-to-face discussion.  Knowledge attitude and practice (NR) |
| Beovic *et al*, 2019^9^ | Multiple countries,  NR | Young medical doctors (n=2366) | Bespoke tool administered via online platform.  Knowledge attitude and practice (48) |
| Bianco *et al*, 2021a^10^ | Italy, Multiple settings | Community Pharmacists (n=415) | Bespoke tool administered via online platform or telephone.  Knowledge attitude and practice (NR) |
| Bianco *et al*, 2021b^11^ | Italy, NR | Dental practitioner (n=563) | Bespoke tool administered via online platform or telephone.  Attitude and practice (NR) |
| Bouchoucha *et al*, 2021^12^ | Australia, University | Nursing students (n=321) | Bespoke tool administered through email  Knowledge (NR) |
| Bounou *et al*, 2021^13^ | Greece, University | Medical (sixth year) and Nursing (fourth year) Student (n=243) | Bespoke tool administered face to face.  Knowledge attitude and practice (32) |
| Briquet *et al*, 2023^14^ | Belgium, Teaching Hospital | Qualified pharmacists, physicians, and nurses. (n=400) | Adopted from ECDC questionnaire administered online through surveyManager.  Knowledge attitude and practice (43) |
| Buckel *et al*, 2016^15^ | USA, Community | Administrators, Pharmacists, and Prescribers (n=588) | Bespoke tool administered online via REDCap  Knowledge and attitude (48) |
| Bunting *et al*, 2020^16^ | USA, NR | Physician's assistant (n=461) | Bespoke tool administered online through QualtricsXM  Knowledge and attitude (21) |
| Carlsson *et al*, 2023^17^ | Sweden, Primary Care | Physicians and Nurses (n=159) | Bespoke tool sent through ordinary mail  Knowledge and attitude (NR) |
| Cataldi *et al*, 2022^18^ | USA, NR | Paediatricians (n=297) | Bespoke tool administered through QualtricsXM or sent by mail  Knowledge attitude and practice (NR) |
| Catton *et al*, 2023^19^ | UK. Hospitals | Consultants, pharmacists and non-consultant level doctors (n=87) | Bespoke tool sent via email  Practice (16) |
| Clemence *et al*, 2018^20^ | Australia, Hospitals | Doctors (n=58) | Bespoke tool administered through SurveyMonkey  Practice (NR) |
| Corrente *et al*, 2021^21^ | Italy, Multiple settings | Veterinary College and High School students, Vet students (n=106) | AMR Eurobacter Survey given during the lesson.  Knowledge attitude and practice (10) |
| Di Gennaro *et al*, 2020^22^ | Italy, Hospitals | Young medical doctors (n=1055) | Bespoke tool administered through SurveyMonkey and promoted in social media and email  Knowledge attitude and practice (19) |
| Evans *et al*, 2019^23^ | Canada, Hospitals | Critical care physicians (n=14) | Bespoke tool administered through email via SurveyMonkey  Attitude (NR) |
| Foote *et al*, 2016^24^ | Canada, University | Undergraduate nursing students  (n=306) | Bespoke tool administered face to face  Practice (NR) |
| Galanis *et al*, 2021^25^ | Greece, Hospitals | HCP (n=106) | The existing Healthcare-Associated Infections questionnaire  Knowledge and practice (NR) |
| Golding *et al*, 2022^26^ | UK, University | Undergraduate veterinary students  (n=573) | Bespoke tool administered through Qualtrics  Knowledge and attitude (NR) |
| Green *et al*, 2018^27^ | New Zealand, Hospitals | Senior clinicians and Managers  (n=122) | Bespoke tool administered through email via SurveyMonkey  Attitude and practice (33) |
| Hamidi *et al*, 2023^28^ | USA, NR | Nurses, (n=78) | Adopted from Merrill et al, 2019^29^ administered via email through MYNANN community  Knowledge attitude and practice (27) |
| Hamilton *et al*, 2020^30^ | USA, NR | Nurse Practitioners, (n=194) | Bespoke tool administered through Qualtrics  Knowledge and attitude (54) |
| Hammerschmidt *et al*, 2019^31^ | Germany, Nursing home | Nurses and Nursing Managers.  (n=165) | Knowledge attitude and practice (23 main questions, five of which had a total of 34 subcategories) |
| Hammoud *et al*, 2022^32^ | Hungary, Hospitals | Nurses, (n=566) | The existing and modified infection control standardised questionnaire (ICSQ) administered face to face  Attitude (NR) |
| Hanna *et al*, 2019^33^ | UK, University | Pharmacy students, (n=112) | Bespoke paper based questionnaire  Knowledge and attitude (NR) |
| Harris *et al*, 2019^34^ | USA, Tertiary care | Physicians who work in ambulatory settings, (n=323) | Bespoke tool administered via email or social medica promotion  Knowledge attitude and practice (34) |
| Hubber *et al*, 2020^35^ | USA, Multiple settings | HCP, (n=86) | Bespoke tool distributed through local provider and email  Knowledge attitude and practice (36) |
| Hurley *et al*, 2023^36^ | USA, Multiple settings | Physician, (n=632) | Bespoke tool administered through Qualtrics and distributed via email and ordinary mail.  Knowledge attitude and practice (NR) |
| Inacio *et al*, 2017^37^ | UK, University | MPharm students in the first, second, third and fourth years,  (n=185) | Bristol Online Services tool distributed through email  Knowledge and attitude (51) |
| Jones *et al*, 2018^38^ | UK, Multiple settings | All dental practitioners completing foundation trainings (3yrs after qualification), or Longitudinal Foundation Training, (n=71) | Bespoke paper-based questionnaire  Knowledge attitude and practice (NR) |
| Jones *et al*, 2020^39^ | UK, Primary care | GPs, nurses, pharmacists, and other healthcare professionals, (n=2373) | The self-assessment tool (SAT) accessible digitally  Practice (16) |
| Kalu *et al* , 2023^40^ | USA, Hospitals | Neonatology fellowship trainees.  (n=139) | Bespoke tool administered email link  Knowledge and attitude (13) |
| Kirk *et al,* 2016^41^ | Multiple countries, Hospitals | Nurses and Physicians, (n=350) | Bespoke online tool administered through email  Knowledge attitude and practice (32) |
| Kiss *et al*., 2019^42^ | Australia, Multiple settings | Infectious Disease physicians, (n=130) | Bespoke tool administered via email through SurveyMonkey  Attitude (NR) |
| Kistler *et al*, 2017^43^ | USA, Nursing home | NH nurses, (n=97) | Paper based survey tool  Knowledge and attitude (NR) |
| Kochlamazashvili *et al*, 2018^44^ | Georgia,  Multiple settings | Doctor of Dental Medicine (DMD), nurses, and dental residents, (n=196) | Bespoke tool  Knowledge attitude and practice (NR) |
| Kufel *et al*, 2020^45^ | USA, Multiple settings | Physicians, resident physicians, and advanced practice providers (nurse practitioners and physician assistants). (n=70) | Bespoke tool distributed via email and administered through Qualtrics  Knowledge attitude and practice (28) |
| Lagadinou *et al*, 2023^46^ | Greece, Hospitals | Healthcare workers, (n=200) | Bespoke survey tool  Knowledge and attitude (NR) |
| Lebentrau *et al*, 2017^47^ | Germany, Hospitals | General surgeons, internists, gynaecologists, and urologists,  (n=456) | Bespoke survey tool  Knowledge and attitude (35) |
| Lee *et al*, 2023^48^ | USA, NR | Community Pharmacists, (n=61) | Bespoke tool developed as a google form  Attitude and practice (15) |
| Levin *et al*, 2019^49^ | France, Multiple settings | Junior physicians, (n=641) | Bespoke tool administered through One Click Survey® and distributed via email  Knowledge attitude and practice (49) |
| Li *et al*, 2021^50^ | Australia, Multiple settings | Nursing staff, (n=430) | Bespoke tool administered via REDCap  Knowledge and attitude (NR) |
| Llor *et al*, 2022^51^ | Spain, Primary care | GP, (n=1107) | Bespoke tool distributed through email  Attitude and practice (16) |
| Loume *et al*, 2023^52^ | Switzerland, Multiple settings | Dentist, (n=92) | Bespoke tool distributed through email  Practice (NR) |
| Macintosh *et al*, 2020^53^ | USA, NR | NICU nurses, (n=188) | Bespoke tool, electronic and paper versions accessible from email, tablet/phone and paper.  Knowledge and attitude (18) |
| Marta-Costa *et al*, 2021^54^ | Portugal, University | Undergraduate students, PhD students, researchers, lecturers, technicians and others, (n=449) | Bespoke tool distributed through email  Knowledge and practice (28) |
| Mazinska *et al*, 2017^55^ | Poland, NR | Physicians, (n=579) | Bespoke tool handed after an event  Knowledge attitude and practice (NR) |
| McCarthy *et al*, 2020^56^ | USA, NR | Dentists and medical providers, (n=156) | Bespoke tool distributed through email  Knowledge and attitude (NR) |
| Mclelland *et al*, 2022^57^ | Australia, University | Veterinary students, (n=90) | Bespoke tool distributed through email and administered via REDCap  Knowledge and attitude (76) |
| Menard *et al*, 2022^58^ | France, Primary care | General public and General practitioners, (n=338) | Bespoke tool  Knowledge and attitude (NR) |
| Merrill *et al*, 2019^29^ | USA, Hospitals | Nurses (n=343) | Bespoke tool distributed through email and administered via Qualtrics  Knowledge attitude and practice (27) |
| Mitchel *et al*, 2021^59^ | Australia, Multiple settings | Nurses and midwives, (n=96) | Bespoke tool distributed through email, advertisement and social media promotion  Knowledge and practice (NR) |
| Montebello *et al*, 2023^60^ | USA, University | Veterinary dentists, (n=104) | Bespoke tool distributed through email and administered via Qualtrics  Knowledge attitude and practice (30) |
| Moriceau *et al*. 2016^61^ | France, Hospitals | Physicians, (n=111) | Bespoke tool created in google drive and distributed through email  Knowledge and attitude (NR) |
| Nelson *et al*, 2017^62^ | USA, NR | Primary care physicians, (n=704) | Bespoke tool distributed through email and ordinary mail (paper version)  Knowledge attitude and practice (NR) |
| Padigos et al, 2020^63^ | New Zealand, Multiple settings | Registered nurses, (n=298) | Bespoke tool distributed through email and administered via Qualtrics  Knowledge and attitude (56) |
| Papini *et al*, 2022^64^ | Italy, Multiple settings | HCW, (n=2137) | Bespoke tool distributed through email and administered via SurveyMonkey  Attitude and practice (24) |
| Peres *et al*, 2016^65^ | Portugal, University | Fifth- and sixth-year students and junior doctors,  (n=223) | Bespoke tool distributed through email  Knowledge (NR) |
| Perozzielo *et al*, 2019^66^ | France, Hospitals | Prescribers,  (n=918) | Paper based questionnaire distributed face to face  Knowledge attitude and practice (NR) |
| Pettke *et al,* 2017^67^ | Germany, Hospitals | HCP and patients, (n=106) | Paper based questionnaire distributed face to face  Knowledge attitude and practice (NR) |
| Peytreman *et al*, 2020^68^ | Switzerland, Primary care | Physicians,  (n=112) | Bespoke tool distributed through email and administered via REDCap  Practice (NR) |
| Platace *et al*, 2016^69^ | Latvia, Hospitals | Infection control specialists and nurses. (n=230) | Bespoke online questionnaire  Attitude (NR) |
| Posada *et al*, 2021^70^ | USA, Dental clinic | Dental health care professionals  (n=265) | Bespoke online questionnaire distributed via email, social media promotion and face to face at a conference  Knowledge attitude and practice (42) |
| Rabano-Blanco *et al*, 2019^71^ | Spain, University | Nursing students, (n=578) | Bespoke tool  Knowledge and attitude (NR) |
| Ricco *et al,* 2022 a^72^ | Italy, Hospitals | Medical professionals  (n=163) | Google form questionnaire distributed through emai  Knowledge attitude and practice (NR) |
| Ricco *et al*, 2022 b^73^ | Italy, NR | Medical professionals, (n=254) | Google form questionnaire distributed through email  Knowledge attitude and practice (NR) |
| Ricco *et al*, 2022c^74^ | Italy,  NR | GP,  (n=157) | Google form questionnaire distributed through email  Knowledge and attitude (NR) |
| Ricco *et al*, 2019^75^ | Italy, Multiple settings | Obstetrics-gynaecologists,  (n=68) | Google form questionnaire distributed through email  Knowledge and attitude (26) |
| Rodrigues *et al*, 2021^76^ | Portugal, Hospitals | Primary care physicians, (n=545) | Questionnaire delivered by post or face to face  Knowledge and attitude (>26) |
| Saha *et al*, 2020^77^ | Australia, Multiple settings | General Practitioners, (n=386) | Paper based questionnaire delivered physically  Knowledge attitude and practice (NR) |
| Saha *et al*, 2021^78^ | Australia, Community | General Practitioners, (n=613) | Paper based questionnaire delivered physically  Knowledge attitude and practice (42) |
| Sahai *et al*, 2016^79^ | Canada, University | Medical residents, (n=89) | Beke questionnaire administered face to face  Knowledge attitude and practice (NR) |
| Sakeena *et al*, 2021^80^ | Australia, University | Pharmacy students, (n=525) | Bespoke tool distributed through email and administered via REDCap  Knowledge and practice (NR) |
| Salm *et al*, 2018^81^ | Germany, NR | GP, (n=340) | Paper questionnaires send via post  Knowledge and practice (32) |
| Salsgiver *et al*, 2018^82^ | USA, Hospitals | Prescribers, (n=402) | Bespoke tool distributed through email and administered via SurveyMonkey  Knowledge attitude and practice (49) |
| Saveanu *et al*, 2022^83^ | Romania, University | Dentistry students, (n=207) | No details provided  Knowledge (20) |
| Schneider *et al*, 2018^84^ | Germany,  NR | General practitioners, hospital physicians (ICU or surgery), veterinary practitioners and pig farmers, (n=1789) | Telephone based survey  Knowledge attitude and practice (NR) |
| Schneider *et al*, 2020^85^ | Germany, Hospitals | Anaesthetists, (n=361) | MR2 survey  Knowledge and attitude (NR) |
| Shukla *et al*, 2017^86^ | Canada, Hospitals | Paediatric residents, (n=85) | Online questionnaire distributed through email  Knowledge and practice (NR) |
| Simoes *et al*, 2018^87^ | Portugal, Hospitals | Physicians, (n=30) | Paper questionnaire distributed physically  Knowledge attitude and practice (NR) |
| Smith *et al*, 2017^88^ | Canada, NR | Physicians, (n=336 in first cycle, 351 in second cycle) | Bespoke questionnaire distributed through email  Knowledge attitude and practice (NR) |
| Sobierajski *et al*, 2021^89^ | Poland, University | Medical students, (n=291) | No details provided  Knowledge and attitude (26) |
| Spernovalis *et al*, 2020^90^ | Greece, Multiple settings | Junior doctors, (n=214) | Online questionnaire administered via SurveyMonkey  Knowledge and attitude (20) |
| Steinberg *et al*, 2016^91^ | Canada, Hospitals | Critical care physicians, (n=185) | Online questionnaire distributed via SurveyMonkey  Knowledge attitude and practice (NR) |
| Struzycka *et al*, 2019^92^ | Poland, University | Medical and dental students, (n=752) | Questionnaire distributed face to face  Knowledge and practice (28) |
| Szumska *et al*, 2022^93^ | Poland, Multiple settings | Medical and non-medical personnel, (n=7544) | Questionnaire distributed face to face  Knowledge attitude and practice (NR) |
| Tomczyk *et al*, 2018^94^ | USA, NR | Dentists, (n=437) | Bespoke questionnaire distributed through email and administered through Vovici 6  Practice (22) |
| Torres *et al*, 2022^95^ | USA, University | Dental Student, (n=109) | Electronic (Qualtrics) and paper survey distributed via email  Knowledge and attitude (31) |
| Trautner *et al*, 2017^96^ | USA, Nursing home | licensed and unlicensed personnels, (n=1626) | No details provided  Knowledge (NR) |
| Trucchi *et al*, 2020^97^ | Italy, Hospitals | HCPs, (n=1410) | Questionnaire administered using google drive  Knowledge attitude and practice (NR) |
| Vaillant *et al*, 2019^98^ | France, Multiple settings | Healthcare Workers, (n=8716) | No details provided  Knowledge and attitude (NR) |
| Van Horrik *et al*, 2022^99^ | Netherlands, Primary care | General practitioners and GP residents, (n=99) | Bespoke questionnaire distributed through email (administered via LimeSurvey version 2.6.7) or post  Knowledge and practice (NR) |
| Vazquez *et al*, 2022^100^ | USA, University | ID and critical care fellows and attending physicians. (n=315) | Bespoke questionnaire distributed through email and administered through Qualtrics  Attitude (27) |
| Venugopalan *et al*, 2016^101^ | USA, Community Teaching facility | House staff Physicians, (n=129) | Paper based bespoke questionnaire distributed face to face  Knowledge attitude and practice (75) |
| Vernooy *et al*, 2022^102^ | Canada, Hospitals | Prescribers, (n=440) | Bespoke questionnaire distributed via email or QR code and administered via SurveyMonkey  Knowledge attitude and practice (35) |
| Walaszek *et al*, 2017^103^ | Poland, Hospitals | Students, medical interns and physicians. (n=100) | No details provided  Knowledge and practice (NR) |
| Weier *et al*, 2017^104^ | Australia, University | Final year medical students,  (n=163) | Bespoke online questionnaire distributed via email or telephone  Knowledge and attitude (NR) |
| Weier *et al*, 2018^105^ | Australia, Hospitals | Pharmacists, (n=259) | Bespoke online questionnaire distributed via email link  Knowledge and attitude (NR) |
| Wiese-Possel *et al*, 2023^106^ | Germany, Multiple settings | Medical students, (n=356) | Bespoke questionnaire distributed via email  Knowledge and attitude (NR) |
| Wilcock *et al*, 2016^107^ | UK, Primary care | General practitioners, (n=40) | Bespoke online questionnaire distributed face to face  Attitude (NR) |
| Wilcox *et al*, 2019^108^ | UK,  Hospitals | HCP and pregnant women  (HCP n=199) | Bespoke questionnaire distributed face to face (paper based) or via email (administered through iSurvey)  Knowledge attitude and practice (NR) |
| Woodard *et al*, 2023^109^ | USA,  NR | Physicians and other healthcare workers, (n=542) | Bespoke questionnaire distributed via email and administered through REDCap  Attitude (99) |
| Xenaki *et al*, 2020^110^ | Greece, NR | Educators or health professionals,  (n=781) | Bespoke questionnaire using google form distributed via social media,  Knowledge attitude and practice (31) |
| Zainaghi *et al*, 2023^111^ | Italy, Multiple settings | Intensive-care physicians and nurses,  (n=143) | Bespoke questionnaire using google form  Knowledge attitude and practice (31) |
| Zetts *et al*, 2020^112^ | USA,  Primary care | Physicians (internal, family, and paediatric medicine physicians), (n=1550) | Bespoke questionnaire distributed through email  Knowledge attitude and practice (NR) |
| Zgliczynski *et al*, 2022^113^ | Poland, University | Medical doctors,  (n=504) | The paper-based questionnaire adopted from the ECDC questionnaire  Knowledge attitude and practice (43) |

Table 2: Findings from the included studies

|  | Population groups | Infection control and management (IPCM)  *(Including vaccine and diseases)* | Antimicrobial stewardship (AMS) | Antimicrobial resistance (AMR) and antibiotic use |
| --- | --- | --- | --- | --- |
| KNOWLEDGE | Doctors (Including Physicians, Specialists, residents, Fellows) | -Respondents showed poor knowledge of infection precautions, especially droplet (11.7% correct) and airborne (16.3%) precautions. More residents (86%) than attending physicians (70%) correctly identified multidrug-resistant urinary infections (p = 0.04). ^61^  -Average HH knowledge was 42.2%, highest before patient contact (78.5%) and lowest before (8.9%) and after (6.3%) PPE use. No HH knowledge differences were seen by residency year, gender, or health sciences background, but Canadian-trained residents scored higher than non-Canadian trained.^79^  - Respondents generally had high knowledge, confidence, and vaccine recommendation rates.^2^  - While most obstetricians and gynaecologists were aware of official vaccination recommendations, only 57.4% correctly recalled the appropriate timing for the seasonal influenza (SID) and Tdap vaccines.^75^  - Knowledge about Respiratory Syncytial Virus (RSV) and its prevention through monoclonal antibodies (mAb) was found to be unsatisfactory, with significant gaps evident in understanding its epidemiology. Key factors influencing risk perception included a background in paediatrics (adjusted odds ratio [aOR]: 55.398, 95% confidence interval [95% CI]: 6.796-451.604) and support for future RSV vaccines (aOR: 4.728, 95% CI: 1.999-11.187). Conversely, prior management of RSV cases (aOR: 0.114, 95% CI: 0.024-0.552) and recommending hospitalization for cases (aOR: 0.240, 95% CI: 0.066-0.869) were associated with lower risk perception.^74^  - Participants demonstrated inadequate knowledge regarding West Nile Virus (WNV), showing significant gaps in understanding its epidemiology and clinical features of West Nile Fever (WNF) and West Nile Neuroinvasive Disease (WNND). Most respondents overlooked WNV as a human pathogen compared to SARS-CoV-2, TB, and HIV.^73^  - Knowledge about monkeypox (MPX) was found to be inadequate, with significant gaps in understanding all aspects of the disease. Risk perception indicated that MPX was largely underestimated compared to other pathogens like SARS-CoV-2, TB, HIV, and HBV.^72^  - Less than 50% of respondents correctly recognised that HAV infections are often asymptomatic in young children and that morbidity increases with age.^62^  - Over 60% of paediatricians answered correctly on 6 of 9 true/false questions about measles knowledge, but less than 50% correctly answered questions on pretravel MMR recommendations for preschoolers and isolation precautions.^18^ | -Knowledge of antibiotic stewardship varied among specialist doctors. Urologists were more aware of local resistance patterns (p < 0.001), but only 36.3% correctly identified ciprofloxacin-resistant E. coli rates.^47^  -While 72.9% were familiar with AMS, 76.5% sought additional training, and 93.6% needed access to guidelines.^78^  -Among participating physicians (response rate 47.6%, 30/63), most recognised antibiotic resistance as a global issue, but one-third did not view it as a significant problem in their own hospital.^87^  -There were no differences in antibiotic knowledge assessment scores based on specialty or years of postgraduate training.^101^  -72% reported having an AMS at their institution. While 86% could identify AMS components, 59% were unsure if they had received AMS training during fellowship. Fellowship year did not significantly affect incorrect knowledge responses (p = 0.40).^40^  -Of the 386 responding GPs, 68.9% were familiar with AMS. ^77^ | -More respondents viewed AMR as a problem in the U.S. (94%) than in their own practice (55%), and inappropriate prescribing was seen as a bigger issue in outpatient settings (91%) than in their own practice (37%). Additionally, 60% believed they prescribed antibiotics more appropriately than their peers.^112^  -Physicians exhibited high knowledge of appropriate antibiotic use and AMR, with effective counselling practices overall.^88^  -Ninety-nine percent of respondents acknowledged that AMR is a national issue, but only 63% recognized it as a local problem in their facilities.^34^  -Good perceived knowledge of antibiotics (OR=3.71) and high perceived education (OR=1.70) were independently associated with considering antibiotic adverse events.^49^  -The majority of physicians cited health-related factors for prescribing antibiotics, including optimal recovery (80%), adherence to the latest therapeutic guidelines (70%), and microbiological/epidemiological considerations (63%).^55^  -Out of 313 invited junior doctors, 214 (68.4%) completed the questionnaire, achieving a mean knowledge score of 60%. Confidence was highest in diagnosing infections and selecting dosages, but lowest in modifying treatment and withholding antimicrobials in febrile patients.^90^  - Physicians demonstrated strong knowledge on antibiotics, with over 98% aware of their side effects, ineffectiveness against viruses, the role of misuse in AMR, and the possibility of healthy individuals carrying resistant bacteria. However, only 47% knew that using antibiotics as growth stimulants in EU livestock is illegal. ^113^  - All GPs expressed strong agreement regarding their knowledge and self-confidence in urine diagnostics and the treatment of asymptomatic bacteriuria (ASB). The median knowledge score was 4 out of 6 (IQR 2 to 6).^99^  - The Certification in Intensive Care (CIC) significantly correlated with self-confidence (p < 0.001), self-rated knowledge (p < 0.001), and objective knowledge (p = 0.029) regarding antibiotic prescription. Senior consultant status was associated with self-confidence (p < 0.001) and self-rated knowledge (p = 0.005), but not with objective knowledge. Additionally, working in an Intensive Care Unit (ICU) in the past 12 months was significantly linked to both self-rated knowledge and self-confidence (all p < 0.001). In a logistic regression model, senior consultant status showed no association with any tested influence factors.^85^  - Of the 386 responding GPs, 68.9% were familiar with AMS.^77^  - Responses varied mostly by country of training and specialty, with few differences based on year of training or gender. Country and type of specialization had a significant impact on all five dimensions, with the strongest effects on self-assessed knowledge and perception of antibiotic resistance.^9^ |
|  | Dentists | -The average knowledge score regarding CDC infection control guidelines was 58%, only 39% identified hand hygiene as the key measure for preventing infection spread. 33% were unaware of the CDC guidelines for respiratory hygiene and cough etiquette in dental settings.^70^ |  | - Most respondents (90.0%) acknowledged that antimicrobial resistance is a significant public health threat in the UK, with 78.9% recognising a moderate or greater impact of dental prescribing on resistance emergence.^38^ |
|  | Veterinarians |  |  | - Knowledge of antibiotic resistance and evidence-based practices did not vary significantly between practice sectors or certification duration.^60^ |
|  | Nurses | -Nurses demonstrated high overall awareness and adherence to standard precautions, educating patients and family members primarily on hand and respiratory hygiene. ^32^  -Most nurses and nursing managers understood effective hand hygiene procedures, and hygiene standards and equipment were generally available. practices.^31^  - The majority of NICU nurses (62.5%) knew immunizations should be given to medically stable infants, but only 27% identified recommended 2-month vaccines. Additionally, 61.1% were unaware of current guidelines for the minimum age of immunization, though nurse practitioners were more knowledgeable and comfortable discussing vaccines with families.^53^ | -Most respondents rated their knowledge of antibiotics and AMR as "average," with 71% citing a lack of knowledge about AMS as the main barrier to its integration in clinical practice. Despite the presence of AMS programs in major district health boards in Auckland, 84% were unaware of them.^63^  -While 64.15% of participants were familiar with AS, time constraints and fear of physician pushback were key barriers to engagement.^1^  -A total of 316 staff nurses from three hospitals (15.8% response rate) participated in the survey. Fifty-two percent were unfamiliar with the term "antimicrobial stewardship," yet 39.6% recognised its importance in their healthcare setting.^29^  -Only 39% of participants were very familiar with the term antibiotic stewardship.^28^ | -Nurse practitioners recognised the consequences of inappropriate antibiotic use, with 97% acknowledging it causes resistance and harms patients, while 94% believed optimal use can reduce resistance.^30^ |
|  | Students (including medical, dental, pharmacy and nursing) | -Only 25% of respondents identified the curriculum as their primary source of information. A positive correlation was found between academic year (P = .032) and prior training in IPCM (p = .016) with overall knowledge.^65^  -Nursing students had slightly better overall knowledge compared to medical students (60.4% vs. 57.2%; p = 0.04).^13^  -Students rated their university education as sufficient for cardiovascular diseases (91.3%) more than for ID (72.5%) and felt more confident in their knowledge of cardiovascular diseases (74.38%) compared to ID (53.76%). They also answered more cardiovascular disease-related clinical questions correctly, achieving a mean score of 78%, compared to a mean score of 45% for antimicrobial use questions.^105^  -Most subjects lacked knowledge about the indirect mode of infection control, persistence of HBV, HCV, or HIV, and the availability of rapid tests (p < 0.05). Awareness of post-exposure prophylaxis (PEP) was limited: 32.36% knew about HBV, 25.60% about HCV, and 36.71% about HIV (p < 0.05). Only 50.24% were aware of the recommended testing moments (p = 0.019 by gender, p = 0.752 by year of study), and just 28.01% identified the optimal time to access PEP (p > 0.05).^83^ | -While 44.5% of participants were familiar with the term "Antimicrobial Stewardship," 70.7% felt they had little to no knowledge of its principles.^12^  -No significant gender differences were found; however, students from the United Kingdom and Ireland exhibited significantly greater knowledge and more appropriate attitudes compared to international students (p<0.001). Their mean overall scores were 73.56 (±5.41) for knowledge versus 68.27 (±6.06).^33^ | -The response rate was 32%, with an overall median knowledge score of 7.9. Significant differences were found in knowledge scores based on years of study (p=0.02), particularly between first-year students (7.6) and fourth-year students (8.3). Male students had higher scores (8.4) compared to female students (7.9) (p=0.03). Most students recognised the importance of strong knowledge in antibiotics, microbiology, and infection control for their pharmacy careers, and over 90% agreed that antibiotic resistance will pose a greater clinical problem in the future.^37^  -Students exhibited a low level of knowledge about antibiotics, scoring an average of 4.1 (CI95% = 3.4-4.8), particularly regarding antibiotic resistance.^71^  -A strong consensus emerged among students regarding the urgency of addressing AMR, with 84.4% advocating for immediate action. While over 94.4% could correctly define AMR, knowledge of its impacts, contributing factors, and potential solutions varied. Many students recognised the significant role of livestock producers in perpetuating AMR, citing prophylactic (71.1%) and treatment (56.7%) antibiotic use. However, 37.8% were uncertain about the transmission of AMR from animals to humans. Respondents identified dentists, doctors, veterinarians, and professional organisations as crucial in tackling AMR.^57^  -Knowledge of AMR and AMS was moderate among early-year veterinary students and good among later-year students.^26^  -Senior pharmacy students (n = 274) demonstrated significantly better knowledge of appropriate antibiotic indications compared to junior students (n = 251) (p < 0.05). Overall, pharmacy students exhibited a solid understanding of AMR, with knowledge improving among senior students.^80^  - High school students had more misconceptions about antibiotics compared to veterinary college students, with common beliefs including: i) antibiotics kill viruses (OR 8.4, CI 4.8–14.7, p < 0.001), ii) they are effective against colds and flu (OR 4.6, CI 2.6–8.1, p < 0.001), and iii) antibiotics can be purchased without a prescription (OR 7.3, CI 4.3–12.5, p < 0.001).^21^ |
|  | Pharmacists |  | About 67.9% recognised the importance of antimicrobial stewardship in the community, and 88.5% would participate if given the opportunity.^48^ |  |
|  | HCPs (mixed population and allied HCPs) | - 62.2% HCPs regularly enhanced their knowledge of infection control, with 86.1% applying infection control rules consistently in their practice.^69^  - The mean knowledge score was 4.7/8 (SD: 1.3) for medical healthcare workers and 3.6/8 (SD: 1.4) for non-medical HCWs. Five factors positively correlated with higher knowledge: working in a university hospital [(aOR): 1.41, 95% CI: 1.16-1.70], ages 26-35 years (aOR: 1.43, 95% CI: 1.23-1.60) and 36-45 years (aOR: 1.19, 95% CI: 1.01-1.40), medical professional status (aOR: 3.7, 95% CI: 3.09-4.44), working in an intensive care unit (aOR: 1.28, 95% CI: 1.06-1.55), and receiving training on antimicrobial resistance (aOR: 1.31, 95% CI: 1.16-1.48).^98^  -Nearly 42% were unaware of the prevalence of HBV) and HCV in Georgia. Knowledge of risk factors for HIV, HCV, and HBV transmission was low, with correct response rates of 45.3%, 37.9%, and 34.2%, respectively. Additionally, 59.7% were uninformed about post-exposure prophylaxis for HIV, and only 37.3% felt well-informed on infection control guidelines.^44^  - Among participants, 26.4% had excellent knowledge about HPV, 44.7% good, 23.6% moderate, and 4.7% each had poor or very poor knowledge. Physicians (80.32%) and educators (65.31%) showed the highest levels of excellent knowledge. A significant proportion (65.8%) were aware of the importance of annual Papanikolaou tests and gynaecological exams.^110^  - The median knowledge and attitude scores regarding HPV were 69.2% (IQR 61.5-84.6) and 5 (IQR 4-5), respectively, with significant differences between physicians and other healthcare professionals. The median propensity score remained stable and high at 10 (IQR 9-10) before and after the educational intervention.^97^  - Sixty percent of caretakers and 59% of staff answered all four knowledge questions correctly.^67^  - Among 159 emergency nurses, 110 emergency physicians, and 161 paramedics, 67.3-78% rated their COVID-19 knowledge as 'good to very good'. The primary source of information was state health department websites.^50^  - The average knowledge score among respondents was 65%. Previous education, training, or practice regarding coccidioidomycosis was the sole predictor of confidence and risk consideration.^35^  - Multivariate linear regression results indicated that participants aware of the infection control program and its hand hygiene protocol had higher knowledge scores regarding hospital infections. Conversely, those who believed their hospital had effective infection control measures had lower knowledge levels. Healthcare professionals who washed their hands before and after patient care, during their shifts, and after removing disposable gloves demonstrated greater knowledge of hospital infections.^25^  - Physician assistants (PAs) in primary care reported more accurate knowledge and higher confidence about HIV. However, knowledge and confidence gaps were present among all PAs, regardless of specialty.^16^  - HCWs exhibited a lack of awareness regarding the links between influenza, vaccination, and cardiovascular health.^8^ | -Most respondents were familiar with antimicrobial stewardship and recognised its necessity.^15^  -Prescribers identified two primary objectives of antibiotic stewardship programs (ASPs): limiting the spread of resistance (77.8%) and improving patient care (76.1%). Most prescribers recognised local guidelines (75.4%) but opposed measures that compromised their autonomy, such as automatic stop orders (23.4%) and pre-approval by advisors (28.8%).^66^  -Ten percent of providers were unfamiliar with antibiotic stewardship, yet 84.3% expressed interest in further AMS education. Notably, 57.1% indicated that pharmacists were valuable resources for promoting appropriate antibiotic prescribing.^45^  -While 99.1% of licensed personnel recognised asymptomatic bacteriuria, only 36.1% knew that pyuria does not differentiate it from a urinary tract infection.^96^ | -While all providers agreed that antibiotics are overprescribed and contribute to resistance, only 42.9% recognised that 90% to 98% of rhinosinusitis cases are viral, and just 5.7% recommended supportive care without antibiotics.^45^  - Overall, 97.8% of respondents from various professions had a good perception of antibiotic resistance. However, only 77.2% felt they had sufficient knowledge, with 91.3% of physicians, 71.5% of nurses, and 63.0% of pharmacists reporting confidence in their capabilities.^14^  - Among respondents, 17% (n = 75) demonstrated knowledge gaps regarding antibiotic resistance and appropriate antibiotics for various infections. All groups agreed that AMR is a major public health issue and acknowledged that antibiotics should only be used for bacterial infections and completed as prescribed (p = 0.00). Groups B and C had significantly better knowledge of antibiotic names and active ingredients compared to undergraduate students (p = 0.00). Group B was notably less likely to have used antibiotics on their pets (p = 0.00). Additionally, groups A, C, and D showed significant awareness of the requirement for a veterinary prescription, while groups C and D were notably aware of the transmission of bacteria between animals and humans.^54^  - Cluster 3 had the highest knowledge of antibiotic use and resistance, cluster 1 showed the greatest awareness of hand hygiene's role in infection prevention.^5^  -Farmers demonstrated three times better basic knowledge of AMR and reported knowing twice as many individuals with multidrug-resistant organism issues compared to the general public. They also received information on AMR more frequently from their veterinarians than patients did from their doctors.^84^  -Around 90% were aware of antibiotic resistance and had access to relevant guidelines and materials. However, many, especially nurses, were unaware of national AMR plans (62.9%) and international campaigns (80%).^111^  -While 97% correctly identified key facts about antibiotic use, fewer than 80% knew that antibiotic use raises a patient's risk of resistant infection or that resistant bacteria can spread person-to-person.^4^  Knowledge of antibiotics (97%) was higher than understanding of antibiotic resistance (75%).^3^  - Knowledge gaps were identified, such as 15% failing to recognise treatment indications for asymptomatic bacteriuria and 59% selecting unnecessarily broad antibiotics based on microbiology susceptibility results. Notably, prescribers' self-reported confidence did not align with their knowledge scores.^102^  - Knowledge gaps were identified through case-based questions, revealing that 29% of respondents sometimes selected overly broad therapy for given susceptibilities, while 32% preferred the most broad-spectrum empiric antimicrobials.^82^  - Half of the respondents understood that antibiotics only target bacteria, while 38% correctly defined antibiotic resistance.^58^  - Among 31 NH nurses and 66 community-dwelling older adults, 70% acknowledged the dangers of antibiotics.^43^  - The survey revealed that both physicians' and nurses' actual knowledge about appropriate antibiotic prescribing was incomplete.^17^ |
| ATTITUDE | Doctors (Including Physicians, Specialists, residents, Fellows) | -90.2% of physicians were vaccinated, and 46.7% (56/120) estimated their staff's vaccination coverage exceeded 60%, despite all practices offering it.^79^  - Only 65.4% expressed a favourable or highly favourable attitude toward a hypothetical WNV vaccine. Higher risk perception of WNV was linked to several factors, including having over 10 years of experience ([aOR] 2.39, 95% Confidence Interval [95% CI] 1.34 to 4.28), better knowledge scores (aOR 2.92, 95% CI 1.60 to 5.30), prior management of WNV cases (aOR 3.65, 95% CI 1.14 to 14.20), support for the hypothetical vaccine (aOR 2.16, 95% CI 1.15 to 4.04), and believing WNV infections could impact daily activities (aOR 2.57, 95% CI 1.22 to 5.42).^73^  - While 58.6% of respondents supported implementing variola vaccination to prevent MPX, those more likely to favour this approach had previously been vaccinated against seasonal influenza ( [aOR] 6.443, 95% Confidence Interval [95% CI] 1.798-23.093) and were supportive of receiving the variola vaccine (aOR 21.416; 95% CI 7.290-62.914).^72^  - While 92% of paediatricians and 59% of family medicine practitioners strongly recommend the HAV vaccine for 1- to 2-year-olds, the belief that the vaccine is required for kindergarten enrolment emerged as the strongest predictor of a physician's recommendation.^62^  - Although generally knowledgeable about RZV, 25% mistakenly believed that side effects from the first dose that interfere with normal activities should prevent receiving the second dose.^36^ | -Many believed AMS could reduce inappropriate antimicrobial use (66.8%) and treatment costs (83.0%).^78^  -57% agreed that an antibiotic approval program improves individual patient care, yet 45% felt it limits their autonomy. Medical residents were more likely than surgical residents to believe the program forces inappropriate antibiotic choices (33% vs. 13%; P=0.02).^101^  -While 91% supported antibiotic stewardship in office-based practices, they ranked antibiotic resistance lower in priority compared to issues like obesity, diabetes, opioids, smoking, and vaccine hesitancy.^112^  -Most critical care physicians (85.7%) believe that handshake stewardship rounds enhance patient care, with only 14.3% considering them an ineffective use of time.^23^  -74% of respondents indicated that their institution had at least one component of an AMS Program. Most (86%) believed that ICU patients benefit from the AMS program, and 81% felt it enhanced their knowledge of appropriate antimicrobial use. Only 11% viewed interactions with the AMS team as time-wasting, while just 7% felt it negatively impacted their autonomy.^91^  -Regression analysis showed that ID physicians valued ICU collaborations more and felt more discomfort with uncertain diagnoses compared to critical care physicians. These factors were linked to stronger agreement that ID physicians should lead ICU antimicrobial stewardship. Higher value of collaboration was associated with female sex, less discomfort with uncertainty, and stronger support for ID stewardship.^100^ | - They were more confident about dosage, frequency, duration (p = 0.038), and interpreting antibiograms (p < 0.001).^47^  - Respondents believed antibiotics are overused nationally more than locally (91% vs. 71%; P=0.0001). While 49% felt other providers overprescribe, only 26% acknowledged their own contribution to the issue (P=0.0003).^101^  -Eighty one percent felt that AMR was inadequately addressed during their medical training, and 71% disagreed that their tutors provided appropriate examples. However, 76% agreed to participate in an active surveillance system or AMS program to combat AMR.^22^  -Counselling on infection prevention topics and proper antibiotic disposal was lower. Those with less than 10 years of experience were significantly less likely to counsel on antibiotic resistance (aOR = 0.27, 95% CI 0.10 to 0.74). In the second-cycle survey, more physicians reported counselling on antibiotic disposal (P = .03) and infection prevention (e.g., antibacterial soap usage, P = .02). Most felt confident in counselling regarding antibiotic use and AMR.^88^  -While 94% understood that each antibiotic prescription affects AMR, 23% believed aggressive prescribing was necessary to prevent clinical failures. The presence of prescription guidelines was perceived to have a low to moderate impact on physicians' antibiotic choices (54%).^34^  -While 93% were aware of bacterial resistance risks, 41% admitted prescribing antibiotics more frequently than necessary.^49^  -A significant 84% were aware of the National Recommendations for the management of Community-Acquired Respiratory Tract Infections (NR-CA-RTI) from the National Programme for Protection of Antibiotics, with 91% of those adhering to them in practice. Physicians primarily sourced their antibiotic prescribing information from Polish medical journals, medical society conferences, and pharmaceutical companies.^55^  -Participants considered guidelines and expert consultations crucial for prescribing practices. Most recognised the antimicrobial resistance issue in Greece, citing excessive and broad-spectrum prescribing as key causes.^90^  - While 98.61% acknowledged the link between antibiotic prescribing and AMR, 65.28% reported a lack of appropriate AMR counselling materials, and 92.5% rarely provided resources on prudent antibiotic use. Notably, 74.4% underestimated the role of hand hygiene in AMR prevention.^113^  - Most GPs (70%, 64 of 92) adhered to guidelines for urine diagnostics and reported appropriate indications for testing. However, 75.5% (71 of 94) would treat patients with diabetes mellitus for ASB, and 37% (34 of 92) would inappropriately repeat a urine test after a patient was treated for a urinary tract infection (UTI). Additionally, one-third of GPs felt that ASB was insufficiently addressed in UTI guidelines.^99^  - Only 57% of residents reported using antibiograms, while general paediatric inpatient attending physicians were recognised as the most influential source of antibiotic knowledge for house staff.^86^  - About, 70.2% (239/340) believed their prescribing behaviour affected local drug resistance. GPs with over 25 years of experience perceived less individual influence on drug resistance compared to those with fewer than 7 years (Odds Ratio [OR] 0.32, 95% Confidence Interval [CI] 0.17-0.62; P < 0.001).^81^  - While 72.9% were familiar with AMS, 76.5% sought additional training, and 93.6% needed access to guidelines. Many believed AMS could reduce inappropriate antimicrobial use (66.8%) and treatment costs (83.0%). CPs frequently counselled patients (97.0%) and reviewed drug interactions (93.8%), but only 45.5% used national Therapeutic Guidelines, and 37.9% assessed compliance. Most CPs supported policies for enhanced GP-pharmacist collaboration (92.4%) and restricting access to certain antimicrobials (74.4%).^78^  - The most commonly used strategies included the Therapeutic Guidelines (TG) at 83.2% and delayed antimicrobial prescribing at 72.2%.^77^  - More than 70% of attitudes differed significantly (p < 0.05) between hospital physicians (HPs) and primary care physicians (PCPs). HPs were more likely to agree that antibiotic resistance is a public health problem and valued microbiological tests and the impact of prescriptions on resistance development. In contrast, PCPs emphasised the negative effects of self-medication with antibiotics obtained without prescriptions and the need for rapid diagnostic tests. Additionally, seven out of nine knowledge sources were deemed more useful by HPs compared to PCPs.^76^ |
|  | Dentists |  |  | -While 90.5% of dentists viewed antibiotic resistance as a concern, only 56.3% felt adequately informed. Many did not follow national guidelines, with inappropriate prescriptions in 11 of 17 clinical situations. Most cited clinical guidelines as the main influence on their prescribing (71%) and expressed a desire for regular guideline updates in practical formats (93%).^6^  -Many felt adequately trained in antimicrobial prescribing, some lacked confidence in issuing prescriptions or performing operative treatments for acute dental conditions. Additional pressures, such as delays in treatment, also affected prescribing practices. A majority (77.5%) advocated for changes to optimize dentists' antimicrobial prescribing, including enhanced availability of clinical guidelines and further undergraduate education.^38^ |
|  | Veterinarians |  |  | -Over 95% of veterinary dentists supported specific antibiotic use guidelines to reduce inappropriate use in veterinary dentistry.^60^ |
|  | Nurses | -There was general awareness of leadership's impact on staff behaviour, not all nursing managers fully recognised the importance of their own consistent role modelling in promoting hand hygiene.^31^  -Nurses and midwives acknowledged the importance of cleaning, but there was confusion about cleaning responsibilities and the appropriate use of disinfectants for rooms of patients with suspected or diagnosed infections post-discharge. Most expressed a lack of confidence in entering a room previously occupied by a patient with a multi-drug-resistant organism.^59^ | -95% of nurses expressed a desire to participate in antimicrobial stewardship interventions.^29^  -The majority agreed that more education is necessary to effectively incorporate antibiotic stewardship principles into practice in the neonatal intensive care unit.^28^ | -94% felt strong antibiotic knowledge was important, and 86% felt confident in their prescribing abilities. Despite this, 94% agreed antibiotics are overused nationally, though only 62% thought the same for their own settings.^30^  -Respondents highlighted the importance of educating patients on safe antibiotic use (98%) and timely administration (97%) as key nursing roles. Younger and less experienced nurses had higher expectations for antibiotic prescriptions for viral illnesses than their older and more experienced counterparts.^63^  - Nurses exhibited more evidence-based attitudes than older adults; however, 39% of nurses expected antibiotics for colds, compared to 28% of older adults.^43^ |
|  | Students (including medical, dental, pharmacy and nursing) | -A significant majority (86.6%) supported mandatory hand hygiene education, and 71.4% considered compulsory education and seminars the most effective measures for improving compliance.^13^  - While most students acknowledge that HPV prevention falls within their scope of practice, 56% expressed limited confidence in both recommending the vaccine and conducting oral cancer examinations.^95^ | -Mean score among the students from the UK and Ireland reported 38.07 (±4.69) for attitudes versus 32.94 (±5.04), with maximum scores of 91 and 45, respectively.^33^  - Only 61.6% (69/112) of respondents felt confident discussing antimicrobial stewardship with patients or other healthcare professionals.^33^ | -Acknowledging knowledge deficiency, over 90% of students advocated for increased training on antibiotics and infection control in the nursing curriculum.^71^  - Among participants, 54% (192/355) strongly agreed that AMR is relevant to clinical practice, and 48% believed their future antibiotic prescribing would impact local AMR development. While students expressed interest in AMR and antibiotic therapy, only 46% correctly answered questions about antibiotic duration for community-acquired pneumonia, and 57% about appropriate antibiotic use for Staphylococcus aureus infections.^106^  -Vet students generally believed they bore less responsibility for causing and preventing AMR compared to other groups, such as animal owners and human medics.^26^  One in four respondents (23.7%) disagreed with antibiotic therapy prescribed by a doctor for their own illness, and 40.9% did so for a family member or friend. Most students (92.4%) expressed a desire to expand their knowledge of antibiotic therapy, but only 20% were aware of the European Antibiotic Awareness Day campaign.^89^ |
|  | Pharmacists |  | -Of the pharmacists surveyed, 78.4% (203/259) reported having an AMS program. Their involvement primarily included assessing total antibiotic consumption and attending AMS committee meetings. Although most pharmacists acknowledged the presence of AMS programs, significant barriers to their engagement were noted in both countries.^105^ |  |
|  | HCPs (mixed population and allied HCPs) | -13.9% reported irregular application, posing risks to both their own health and that of patients. Key motivating factors for adherence to infection control included the importance of rules in patient care (94.3%), availability of guidelines in the ward (92.6%), regular supervision by head nurses (84.8%), positive colleague attitudes (80.4%), and access to necessary equipment and personal protective gear.^69^  -Over half of respondents from both countries agreed that they would be more likely to clean their hands if alcohol-based hand rubs were more accessible near patients.^41^  -95.6% expressed interest in further education on the occupational transmission of blood-borne pathogens.^44^  Most midwives (66%) had little to no awareness of RSV. They were less likely than obstetricians to support clinical trials (68% vs. 92%, OR: 2.50) and routine RSV vaccination (79% vs. 89%, OR: 4.08). Midwives with prior knowledge of RSV and those who viewed it as serious were more likely to support vaccination efforts.^108^  - Predictors of higher knowledge scores about HPV included being a physician, general practitioner, or paediatrician, attending courses or congresses, and consulting scientific literature on the HPV vaccine. Similarly, being a physician and using scientific literature as a resource also predicted significantly different attitude scores among participants.^97^  - Main motivations for vaccination (over 75% in at least one cohort) included disease prevention and concern about transmission (77-100%).^67^  -COVID-19 vaccination hesitancy was more common among females, less concerned individuals, and nurses and healthcare assistants. Hesitant professionals were less likely to recommend vaccination to others, while those with higher concern recommended it more. Most healthcare workers (61.22%) supported mandatory vaccination. Many relied on institutional information, while those in northern Italy and specific specialties preferred scientific literature; workers in south-central regions often turned to the internet and media for information.^64^ | -Participants viewed antibiotic stewardship positively and were most interested in training, audit, and feedback improvements.^7^  -While 78.7% of respondents reported access to local antimicrobial prescribing guidelines, there was frequent discordance in responses from practitioners within the same institution.^27^  -Regarding opportunities, 72.2% had easy access to necessary infection management guidelines, while 64.2% indicated that this information altered their views on inappropriate antibiotic use. Additionally, 55.0% reported changes in their practices due to this information.^14^  -Among unlicensed personnel, 99.6% would notify a nurse about fever or confusion, but only 27.7% understood that cloudy, smelly urine should not be routinely cultured. Despite 100% reporting hand hygiene training, fewer than 30% knew the correct hand-rubbing duration (28.5% licensed, 25.2% unlicensed) or the most effective agent to use (11.7% licensed, 10.6% unlicensed).^96^  - Prescribers preferred educational interventions (73.0%) and clinical staff meetings (70.0%).^66^ | -While many pharmacists and prescribers acknowledged the overuse of antimicrobials at their hospital, SCH pharmacists and prescribers were less likely to view local antibiotic resistance as a significant issue. Pharmacists favoured restrictions on antibiotic use more than prescribers did. Additionally, SCH practitioners were less familiar with IDSA guidelines and relied less on ID specialists compared to LCH practitioners. Most respondents expressed a strong desire for more antimicrobial education.^15^  -Of the 159 pet owners surveyed, half had administered antibiotics to their animals, and 64% (n = 102) recognised the need for a veterinary prescription.^54^  -Cluster 2 most recognised their advisory role in promoting prudent antibiotic use.^5^  -Each group viewed the drivers of AMR as external to their own activities. Guidelines were a crucial information source for antibiotic therapy across all prescriber groups, with varying routine usage: 39% of GPs, 65% of hospital physicians, and 53% of veterinarians.^84^  -Most physicians (70%-90%) trusted their prescribing abilities, followed guidelines, and recognised their role in combating antibiotic resistance.^111^  -While 60% had received information on avoiding unnecessary antibiotic use, only 55% gave advice to patients, and just 17% provided resources.^3^  - All respondents recognised AMR as a significant challenge in Canada, with 86% acknowledging it as a serious problem at their hospital. However, only 36% believed antibiotics are misused locally. Most (92%) agreed that AMS can reduce AMR.^102^  - Although 99% reported reviewing antimicrobial appropriateness at 48-72 hours, only 55% did so "always." Additionally, 45% felt inadequately trained in antimicrobial prescribing. Confidence issues were noted in selecting empiric therapy using antibiograms (30%), interpreting susceptibility results (24%), de-escalating therapy (18%), and determining therapy duration (31%).^82^  - While the practice of prescribing prophylactic antibiotics is common, the factors influencing these decisions are not well understood. Survey revealed significant differences in the factors affecting decision-making and perceived responsibilities regarding prophylactic antibiotic use between the two groups, yet both expressed a strong desire for additional training on the topic.^56^  - Most respondents were Infectious Diseases physicians, microbiologists, or trainees in Australia, with 88% prescribing long-term antibiotics for varied indications like recurrent UTIs and cellulitis. Ninety-five percent would stop long-term therapy if appropriate, and 74% were willing to enrol patients in a trial on this topic.^42^  - A large majority of respondents felt they were well-acquainted with the guidelines but exhibited optimism bias, believing that physicians and nurses generally know and follow the guidelines less effectively than they do. They identified patient expectations as the primary reason for non-compliance.^17^ |
| PRACTICE | Doctors (Including Physicians, Specialists, residents, Fellows) | -Most practices (55.7%) had no specific mask-wearing recommendations. Physicians frequently reported hand hygiene before patient exams (74.6%), after exams (90.2%), and before procedures (91.8%). However, compliance was lower upon arriving at (63.9%) and leaving (68.0%) the practice.^79^  - Male providers and those aged 50 and over recommended the vaccine to boys aged 11-12 less frequently than female and younger providers.^2^  - Overall, 79.4% and 67.6% of participants reported regularly administering or recommending SID and tetanus-diphtheria-acellular pertussis vaccinations (Tdap) to pregnant women, respectively. Significant predictors for Tdap practice included higher risk perception (OR 6.466, 95% CI: 1.077-38.803) and better knowledge of recommendations (OR 7.310, 95% CI: 1.195-44.704), while SID practice did not correlate with participants' individual characteristics.^75^  - Many physicians recommended RZV to immunocompromised populations, including 67% for adults aged 50 and older with HIV and 56% on recombinant human immune modulator therapy. Forty-seven percent both stocked/administered RZV and referred patients elsewhere for vaccination, while 42% only referred patients.^36^  - The most consulted resources for measles information included the American Academy of Paediatrics (72%), state/local health departments (70%), and the CDC (63%). More than 90% reported correct clinical practices for vaccinating a 9-month-old before international travel, yet over one-third lacked a plan for measles exposures in their clinic.^18^ | -Respondents frequently counselled patients (97.0%) and reviewed drug interactions (93.8%), but only 45.5% used national Therapeutic --Guidelines, and 37.9% assessed compliance. Most participants supported policies for enhanced GP-pharmacist collaboration (92.4%) and restricting access to certain antimicrobials (74.4%).^78^ | -The most commonly used strategies included the Therapeutic Guidelines (TG) at 83.2% and delayed antimicrobial prescribing at 72.2%. However, fewer GPs utilised point-of-care tests (18.4%), patient information leaflets (20.2%), peer prescribing reports (15.5%), or audit and feedback (9.8%). GPs were generally receptive to pharmacists' recommendations for antimicrobial choice (50.5%) and dosage (63%), with over 60% supporting increased GP-pharmacist collaboration.^77^  -Urologists prescribed antibiotics to more than five patients within seven workdays more frequently than non-urologists (50.7% vs. 24.3%; p < 0.001).^47^  - Two factors linked to appropriate prescribing were high perceived education on antibiotic use (OR=1.51) and medical specialty (OR=1.69).^49^  - Deviations from clinical guidelines for antibiotic treatments were greater for sinusitis and community-acquired pneumonia than for otitis media and group A streptococcal pharyngitis. ^86^  - While 99.1% (337/340) were aware of the "delayed prescription" strategy to reduce antibiotic use, only 29.4% (74/340) reported using it "often" or "very often," with rural GPs applying it less frequently than their urban counterparts.^81^  - Fewer GPs utilised point-of-care tests (18.4%), patient information leaflets (20.2%), peer prescribing reports (15.5%), or audit and feedback (9.8%). GPs were generally receptive to pharmacists' recommendations for antimicrobial choice (50.5%) and dosage (63%), with over 60% supporting increased GP-pharmacist collaboration.^77^  - A total of 92.5% (95% CI: 90.8-94%) of GPs reported using the antibiotic deprescribing (AD) strategy at least once. Confidence in using AD was high for common cold (97.6%) and influenza (93.5%), but lower for acute bronchitis (45.5%). Only 12.1% (95% CI: 10.2-14.2%) viewed AD as potentially harmful. AD was used more often when the antibiotic course was initiated by the prescribing GP (96.8%) compared to another doctor (52.3%). Older doctors (>60 years) were more likely to use AD than their younger counterparts (64.5% vs. 50%; p < 0.005).^51^  - Out of 274 antibiotic choices, 197 (72%) were deemed appropriate, with 149 (54%) classified as optimal. Antibiotics were more likely to be chosen appropriately when based on the Therapeutic Guidelines rather than current hospital practices (p = 0.02). There was no significant difference in appropriateness based on the doctor's grade (p = 0.34).^20^  - Over half (54.2%) of young doctors prescribed antibiotics based on mentor guidance.^9^ |
|  | Dentists |  |  | - Among 437 dentists surveyed, the median monthly antibiotic prescriptions included 4 for prophylaxis and 5 for treatment. Common prophylaxis reasons included "high-risk conditions" (84%) and localised swelling (70%), but inappropriate factors such as upcoming vacations (38%) and gingival pain (38%) were also cited. Overall, dentists reported higher antibiotic use than current guidelines recommend.^94^  - Antibiotics were mainly used for abscesses with systemic symptoms (89%), cellulitis (81.5%), and acute sinusitis (62%). Common procedures paired with antibiotics were sinus floor elevation (59.8%) and implant placement (60.9%). Most dentists recommended prophylaxis for endocarditis risk (82.6%) and immunodeficiency (50%). Additionally, 76.1% expressed a need for clearer antibiotic guidelines in dentistry.^52^  - Out of 563 dental practitioners, antibiotic prophylaxis (AP) prescribing in line with guidelines. However, AP was unnecessarily prescribed for healthy patients in 41.9% of luxation injuries and 70.3% for bone grafting. In 70.9% of procedures, AP courses did not follow guidelines, indicating a high rate of unnecessary AP prescriptions before dental procedures.^11^ |
|  | Veterinarians |  |  | - Dentists (veterinary dentists) who doubted the effectiveness of postoperative antibiotics were less likely to prescribe them intraoperatively.^60^ |
|  | Nurses | -Those with higher awareness provided more education to patients, with significant differences noted only for respiratory hygiene and the rationale for isolation.^32^  -Compliance varied based on availability in the immediate work area and role modelling.^31^ |  | - A significant majority of nurses did not perceive the need for antibiotics in three vignettes: 77% for ASB, 87% for URI, and 97% for a wound.^43^ |
|  | Students (including medical, dental, pharmacy and nursing) | -Medical students reported better hand hygiene practices than nursing students (69.9% vs. 59.7%; p < 0.001).^13^  -Overall, 74.8% of students were deemed hand hygiene compliant, with compliance exceeding 90% both before and after direct patient contact.^24^  -Only 37.68% strongly agreed that the one-hand covering technique for needles was effective in infection control (p < 0.05).^83^ |  | -The majority of participants (88%) reported using antibiotics in the past year . Junior pharmacy students were more likely to incorrectly recommend antibiotics for cold and flu management (p < 0.05).^80^  -About 54% of respondents believed dentists overprescribe antibiotics, with 7% thinking antibiotics are effective for the flu and 11% for the common cold. Amoxicillin (46%) and clindamycin (44%) were identified as the first-choice treatments for dentoalveolar abscesses.^92^  - Fifty-five percent of veterinary college students and 79% of high school students reported taking antibiotics in the past year.^21^ |
|  | Pharmacists |  |  | About 37.1% of community pharmacists participated in dispensing antibiotics without prescription, despite 93.7% knowing it is illegal in Italy. Most CPs regularly asked about drug allergies (95.5%) and medication history (82.5%), while 66.2% warned about side effects, and 55% emphasised completing the full antibiotic course.^10^ |
|  | HCPs (mixed population and allied HCPs) | -Infection rounds varied in frequency, with 10% offering only phone advice. Antibiotic guidance was available in 99% of units, but only 8% was specific to ICUs. There were differences in the availability of biomarkers and the duration of antibiotics prescribed for pneumonia, urinary infections, intra-abdominal infections, and sepsis. Antibiotic consumption data were not routinely discussed in multidisciplinary meetings.^19^  -Proper HH was observed in 64.7% (4,879) of subjects, while 35.3% (2,665) disinfected incorrectly. Nurses demonstrated better hand hygiene than physicians, especially in general departments (62.1% vs. 69.2%; p = 0.0019). Overall, inadequate hand hygiene practices persist among some hospital workers.^93^  -About 75% of physicians and students did not correctly follow HH techniques. Respondents reported performing HH in the following situations: 74.4% before aseptic tasks, 60.8% before patient contact, 57.0% after patient contact, 11.5% after exposure to body fluids, and only 1.1% after contact with patient surroundings.^103^  -Despite strong support for vaccinating boys and girls against HPV (94.1%), many participants were not vaccinated themselves (74.8%) or had not vaccinated their children (19.7%).^110^  - Pharmacists were less likely than MD/DOs to intend to get vaccinated (COVID-19) immediately (75.0% vs 91.4%, p<0.001) and to encourage patients to get vaccinated (78.6% vs 91.0%, p=0.01). Providers in suburban areas were less likely to recommend vaccines to patients (OR=0.43) and family (OR=0.45) compared to urban providers. Those over 45 were more likely to intend to get vaccinated early (OR=3.72).^109^  - Among respondents, 32% of staff and 54% of caretakers had a history of influenza, while 61% of staff and 47% of caretakers had received at least one influenza vaccination. Vaccination rates last season were 47% for staff, 34% for primary caretakers, 30% for partners, 25% for diseased children, and 29% for siblings. Reasons for not vaccinating included fear of adverse effects and reliance on alternative protections (33-83%). Significant associations with vaccination included past vaccinations (OR 2.2-20.5), healthcare provider recommendations (OR 4.8-45.5), lower education levels among caretakers (OR 2.2), and younger age among children (OR 0.9).^67^  - Participants rarely discussed the benefits of influenza vaccination or recommended it to patients, likely due to a combination of limited awareness, perceiving it as outside their responsibilities, and workload challenges.^8^ | -The median implementation score for core ABS elements was 3.3, with no association to nursing home characteristics.^7^  -Clinicians reported high engagement in positive AMS practices, including using antibiotic guidance for treatment decisions (98%), discussing antibiotic prescribing within the practice (73%), utilising patient-facing resources (94%), conducting antibiotic audits in the past two years (98%), maintaining written records and action plans (81%), employing backup prescribing (99%), and implementing clinical coding (80%).^39^ | -Although 81% linked their prescribing behaviour to the spread of resistance, only 64% felt responsible for controlling it. Around 35% of UK prescribers had prescribed antibiotics in the prior week due to concerns about patient deterioration.^4^  -Fear of patient deterioration or complications (43%) was the main reason for unnecessary antibiotic prescriptions.^3^  -Healthcare workers largely avoid prescribing antibiotics based on patient opinion, with 97% aware of their main adverse effects. However, 25% prescribe due to diagnostic uncertainty, and 32.5% rely on experience. HCWs rarely report adverse effects, and inappropriate prescribing is seen as a key driver of bacterial resistance. Post hoc analysis revealed that nursing staff are less likely than consultants, doctor directors, and trainees to follow national guidelines (p: 0.011) and use electronic prescriptions (p: 0.003).^46^  - Twenty-seven percent of respondents reported taking antibiotics in the past year. Sixty-five percent of GPs indicated they had prescribed fewer antibiotics over the last five years, although 33% noted that patients often pressured them for prescriptions, particularly elderly patients with comorbidities.^58^ |

**Section 3:** Quality appraisal of the included studies

|  | External Validity | | | | Internal Validity | | | | | |  |  |
| --- | --- | --- | --- | --- | --- | --- | --- | --- | --- | --- | --- | --- |
| Study ID (Author, year) | Q1. | Q2. | Q3. | Q4. | Q5. | Q6. | Q7. | Q8. | Q9. | Q10. | Score | Overall RoB |
| Abbas *et al*, 2019^1^ | 1 | 0 | 1 | 0 | 0 | 0 | 1 | 0 | 0 | 0 | 3 | low |
| Alcala *et al*, 2018^2^ | 1 | 0 | 1 | 1 | 0 | 0 | 1 | 0 | 0 | 0 | 4 | moderate |
| Ashiru *et al*, 2021^3^ | 0 | 0 | 0 | 0 | 0 | 0 | 0 | 1 | 0 | 0 | 1 | low |
| Ashiru *et al*, 2022^4^ | 0 | 0 | 0 | 0 | 0 | 0 | 0 | 1 | 0 | 1 | 2 | low |
| Barchitta *et al*., 2021^5^ | 0 | 0 | 0 | 0 | 0 | 1 | 1 | 1 | 0 | 1 | 4 | moderate |
| Baudet *et al*., 2020^6^ | 0 | 0 | 0 | 1 | 0 | 1 | 1 | 0 | 0 | 0 | 3 | low |
| Belan *et al*., 2021^7^ | 1 | 0 | 1 | 1 | 0 | 0 | 1 | 1 | 0 | 0 | 5 | moderate |
| Benedict Kpozehouen *et al*., 2023^8^ | 1 | 0 | 1 | 1 | 0 | 1 | 0 | 1 | 0 | 0 | 5 | moderate |
| Beovic *et al*, 2019^9^ | 0 | 0 | 0 | 1 | 0 | 0 | 0 | 1 | 0 | 1 | 3 | low |
| Bianco *et al*, 2021a^10^ | 1 | 1 | 1 | 0 | 0 | 1 | 1 | 1 | 0 | 0 | 6 | moderate |
| Bianco *et al*, 2021b^11^ | 0 | 0 | 0 | 1 | 0 | 0 | 1 | 1 | 0 | 0 | 3 | low |
| Bouchoucha *et al*, 2021^12^ | 1 | 0 | 1 | 1 | 0 | 0 | 1 | 0 | 0 | 0 | 4 | moderate |
| Bounou *et al*, 2021^13^ | 1 | 0 | 0 | 1 | 0 | 0 | 1 | 1 | 0 | 0 | 4 | moderate |
| Briquet *et al*, 2023^14^ | 1 | 0 | 0 | 1 | 0 | 1 | 1 | 0 | 0 | 1 | 5 | moderate |
| Buckel *et al*, 2016^15^ | 1 | 0 | 0 | 1 | 0 | 1 | 1 | 1 | 0 | 0 | 5 | moderate |
| Bunting *et al*, 2020^16^ | 1 | 1 | 1 | 1 | 0 | 0 | 1 | 0 | 0 | 0 | 5 | moderate |
| Carlsson *et al*, 2023^17^ | 0 | 0 | 0 | 1 | 0 | 0 | 1 | 0 | 0 | 1 | 3 | low |
| Cataldi *et al*, 2022^18^ | 0 | 0 | 1 | 0 | 0 | 0 | 1 | 1 | 0 | 1 | 4 | moderate |
| Catton *et al*, 2023^19^ | 1 | 0 | 0 | 1 | 0 | 1 | 1 | 0 | 0 | 1 | 5 | moderate |
| Clemence *et al*, 2018^20^ | 1 | 1 | 0 | 0 | 0 | 0 | 1 | 0 | 0 | 0 | 3 | low |
| Corrente *et al*, 2021^21^ | 1 | 0 | 1 | 0 | 0 | 0 | 1 | 1 | 0 | 0 | 4 | moderate |
| Di Gennaro *et al*, 2020^22^ | 0 | 0 | 0 | 0 | 0 | 0 | 0 | 1 | 0 | 0 | 1 | low |
| Evans *et al*, 2019^23^ | 1 | 0 | 1 | 1 | 0 | 0 | 1 | 0 | 0 | 1 | 5 | moderate |
| Foote *et al*, 2016^24^ | 1 | 0 | 0 | 1 | 0 | 1 | 1 | 1 | 0 | 0 | 5 | moderate |
| Galanis *et al*, 2021^25^ | 1 | 1 | 0 | 1 | 0 | 1 | 0 | 1 | 0 | 1 | 6 | moderate |
| Golding *et al*, 2022^26^ | 1 | 0 | 1 | 1 | 0 | 1 | 1 | 0 | 0 | 0 | 5 | moderate |
| Green *et al*, 2018^27^ | 1 | 0 | 0 | 0 | 0 | 1 | 1 | 0 | 0 | 0 | 3 | low |
| Hamidi *et al*, 2023^28^ | 0 | 0 | 0 | 0 | 0 | 0 | 1 | 0 | 0 | 0 | 1 | low |
| Hamilton *et al*, 2020^30^ | 1 | 1 | 0 | 1 | 0 | 0 | 1 | 0 | 0 | 1 | 5 | moderate |
| Hammerschmidt *et al*, 2019^31^ | 1 | 0 | 0 | 1 | 0 | 1 | 1 | 1 | 0 | 0 | 5 | moderate |
| Hammoud *et al*, 2022^32^ | 1 | 0 | 0 | 0 | 0 | 1 | 1 | 0 | 0 | 0 | 3 | low |
| Hanna *et al*, 2019^33^ | 1 | 0 | 0 | 0 | 0 | 1 | 1 | 0 | 0 | 0 | 3 | low |
| Harris *et al*, 2019^34^ | 1 | 0 | 1 | 0 | 0 | 1 | 1 | 1 | 0 | 0 | 5 | moderate |
| Hubber *et al*, 2020^35^ | 1 | 0 | 1 | 1 | 0 | 0 | 1 | 0 | 0 | 0 | 4 | moderate |
| Hurley *et al*, 2023^36^ | 0 | 0 | 0 | 0 | 0 | 1 | 1 | 0 | 0 | 0 | 2 | low |
| Inacio *et al*, 2017^37^ | 1 | 0 | 1 | 1 | 0 | 1 | 1 | 0 | 0 | 1 | 6 | moderate |
| Jones *et al*, 2018^38^ | 0 | 0 | 1 | 0 | 0 | 0 | 0 | 0 | 0 | 0 | 1 | low |
| Jones *et al*, 2020^39^ | 0 | 0 | 1 | 1 | 0 | 1 | 1 | 0 | 0 | 0 | 4 | moderate |
| Kalu *et al* , 2023^40^ | 1 | 0 | 1 | 0 | 0 | 1 | 1 | 0 | 0 | 0 | 4 | moderate |
| Kirk *et al,* 2016^41^ | 0 | 0 | 1 | 1 | 0 | 1 | 0 | 0 | 0 | 0 | 3 | low |
| Kiss *et al*., 2019^42^ | 1 | 0 | 1 | 1 | 0 | 1 | 1 | 0 | 0 | 0 | 5 | moderate |
| Kistler *et al*, 2017^43^ | 1 | 0 | 0 | 1 | 0 | 0 | 1 | 0 | 0 | 0 | 3 | low |
| Kochlamazashvili *et al*, 2018^44^ | 1 | 0 | 0 | 1 | 0 | 1 | 1 | 1 | 0 | 0 | 5 | moderate |
| Kufel *et al*, 2020^45^ | 1 | 0 | 1 | 1 | 0 | 1 | 1 | 0 | 0 | 0 | 5 | moderate |
| Lagadinou *et al*, 2023^46^ | 1 | 0 | 0 | 1 | 0 | 0 | 0 | 1 | 0 | 0 | 3 | low |
| Lebentrau *et al*, 2017^47^ | 1 | 0 | 1 | 1 | 0 | 0 | 0 | 1 | 0 | 1 | 5 | moderate |
| Lee *et al*, 2023^48^ | 1 | 0 | 1 | 1 | 0 | 1 | 1 | 0 | 0 | 1 | 6 | moderate |
| Levin *et al*, 2019^49^ | 0 | 0 | 0 | 1 | 0 | 0 | 0 | 0 | 0 | 0 | 1 | low |
| Li *et al*, 2021^50^ | 1 | 0 | 0 | 1 | 0 | 0 | 0 | 1 | 0 | 0 | 3 | low |
| Llor *et al*, 2022^51^ | 1 | 0 | 1 | 1 | 0 | 1 | 1 | 0 | 0 | 0 | 5 | moderate |
| Loume *et al*, 2023^52^ | 1 | 0 | 0 | 1 | 0 | 0 | 1 | 0 | 0 | 0 | 3 | low |
| Macintosh *et al*, 2020^53^ | 1 | 0 | 0 | 1 | 0 | 1 | 1 | 0 | 0 | 1 | 5 | moderate |
| Marta-Costa *et al*, 2021^54^ | 0 | 0 | 1 | 1 | 0 | 0 | 1 | 0 | 0 | 0 | 3 | low |
| Mazinska *et al*, 2017^55^ | 1 | 0 | 1 | 1 | 0 | 0 | 1 | 0 | 0 | 0 | 4 | moderate |
| McCarthy *et al*, 2020^56^ | 1 | 1 | 1 | 1 | 0 | 1 | 1 | 0 | 0 | 0 | 6 | moderate |
| Mclelland *et al*, 2022^57^ | 1 | 0 | 0 | 0 | 0 | 0 | 0 | 0 | 0 | 1 | 2 | low |
| Menard *et al*, 2022^58^ | 0 | 0 | 0 | 1 | 0 | 1 | 1 | 1 | 0 | 1 | 5 | moderate |
| Merrill *et al*, 2019^29^ | 1 | 0 | 0 | 1 | 0 | 0 | 1 | 0 | 0 | 0 | 3 | low |
| Mitchel *et al*, 2021^59^ | 0 | 0 | 0 | 1 | 0 | 0 | 1 | 0 | 0 | 0 | 2 | low |
| Montebello *et al*, 2023^60^ | 1 | 0 | 1 | 1 | 0 | 0 | 0 | 0 | 0 | 0 | 3 | low |
| Moriceau *et al*. 2016^61^ | 1 | 0 | 1 | 1 | 0 | 0 | 1 | 0 | 0 | 0 | 4 | moderate |
| Nelson *et al*, 2017^62^ | 1 | 0 | 0 | 0 | 0 | 0 | 0 | 0 | 0 | 1 | 2 | low |
| Padigos et al, 2020^63^ | 1 | 0 | 0 | 1 | 0 | 1 | 0 | 0 | 0 | 0 | 3 | low |
| Papini *et al*, 2022^64^ | 0 | 0 | 1 | 1 | 0 | 0 | 1 | 0 | 0 | 0 | 3 | low |
| Peres *et al*, 2016^65^ | 1 | 0 | 1 | 1 | 0 | 1 | 1 | 0 | 0 | 0 | 5 | moderate |
| Perozzielo *et al*, 2019^66^ | 1 | 0 | 0 | 1 | 0 | 0 | 1 | 0 | 0 | 0 | 3 | low |
| Pettke *et al,* 2017^67^ | 1 | 0 | 0 | 1 | 0 | 0 | 1 | 0 | 0 | 0 | 3 | low |
| Peytreman *et al*, 2020^68^ | 0 | 0 | 1 | 0 | 0 | 0 | 0 | 1 | 0 | 0 | 2 | low |
| Platace *et al*, 2016^69^ | 1 | 0 | 1 | 1 | 0 | 1 | 0 | 1 | 0 | 0 | 5 | moderate |
| Posada *et al*, 2021^70^ | 0 | 0 | 0 | 0 | 0 | 0 | 0 | 1 | 0 | 0 | 1 | low |
| Rabano-Blanco *et al*, 2019^71^ | 1 | 0 | 1 | 0 | 0 | 0 | 1 | 1 | 0 | 0 | 4 | moderate |
| Ricco *et al*, 2022a^72^ | 1 | 0 | 0 | 1 | 0 | 0 | 1 | 0 | 0 | 0 | 3 | low |
| Ricco *et al*, 2022b^73^ | 1 | 1 | 1 | 1 | 0 | 0 | 0 | 1 | 0 | 0 | 5 | moderate |
| Ricco *et al*, 2022c^74^ | 1 | 0 | 1 | 1 | 0 | 0 | 0 | 1 | 0 | 0 | 4 | moderate |
| Ricco *et al*, 2019^75^ | 1 | 0 | 0 | 0 | 0 | 0 | 0 | 1 | 0 | 0 | 2 | low |
| Rodrigues *et al*, 2021^76^ | 1 | 0 | 1 | 1 | 0 | 0 | 0 | 1 | 0 | 0 | 4 | moderate |
| Saha *et al*, 2020^77^ | 0 | 0 | 0 | 1 | 0 | 1 | 1 | 1 | 0 | 0 | 4 | moderate |
| Saha *et al*, 2021^78^ | 0 | 0 | 1 | 1 | 0 | 1 | 0 | 1 | 0 | 0 | 4 | moderate |
| Sahai *et al*, 2016^79^ | 1 | 0 | 0 | 1 | 0 | 0 | 0 | 1 | 1 | 0 | 4 | moderate |
| Sakeena *et al*, 2021^80^ | 1 | 0 | 0 | 1 | 0 | 1 | 0 | 0 | 0 | 0 | 3 | low |
| Salm *et al*, 2018^81^ | 1 | 0 | 0 | 1 | 0 | 0 | 0 | 0 | 0 | 1 | 3 | low |
| Salsgiver *et al*, 2018^82^ | 1 | 0 | 1 | 1 | 0 | 1 | 1 | 0 | 0 | 0 | 5 | moderate |
| Saveanu *et al*, 2022^83^ | 1 | 0 | 0 | 1 | 0 | 1 | 0 | 1 | 0 | 0 | 4 | moderate |
| Schneider et al, 2018^84^ | 1 | 0 | 1 | 1 | 0 | 1 | 1 | 0 | 0 | 1 | 6 | moderate |
| Schneider et al, 2020^85^ | 1 | 0 | 1 | 1 | 0 | 1 | 1 | 1 | 0 | 0 | 6 | moderate |
| Shukla *et al*, 2017^86^ | 1 | 0 | 1 | 1 | 0 | 1 | 1 | 0 | 0 | 0 | 5 | moderate |
| Simoes *et al*, 2018^87^ | 1 | 0 | 0 | 1 | 0 | 0 | 1 | 0 | 0 | 0 | 3 | low |
| Smith *et al*, 2017^88^ | 0 | 0 | 1 | 1 | 0 | 1 | 0 | 1 | 0 | 0 | 4 | moderate |
| Sobierajski *et al*, 2021^89^ | 1 | 0 | 0 | 1 | 0 | 0 | 0 | 1 | 0 | 0 | 3 | low |
| Spernovalis *et al*, 2020^90^ | 1 | 0 | 0 | 0 | 0 | 0 | 0 | 0 | 0 | 0 | 1 | low |
| Steinberg *et al*, 2016^91^ | 1 | 0 | 0 | 1 | 0 | 1 | 1 | 0 | 0 | 0 | 4 | moderate |
| Struzycka *et al*, 2019^92^ | 1 | 0 | 0 | 0 | 0 | 1 | 1 | 1 | 0 | 1 | 5 | moderate |
| Szumska *et al*, 2022^93^ | 1 | 0 | 0 | 1 | 0 | 1 | 1 | 0 | 0 | 0 | 4 | moderate |
| Tomczyk *et al*, 2018^94^ | 1 | 1 | 1 | 1 | 0 | 1 | 0 | 1 | 0 | 0 | 6 | moderate |
| Torres *et al*, 2022^95^ | 1 | 0 | 0 | 1 | 0 | 0 | 1 | 0 | 0 | 0 | 3 | low |
| Trautner *et al*, 2017^96^ | 1 | 0 | 0 | 0 | 0 | 1 | 1 | 1 | 0 | 0 | 4 | moderate |
| Trucchi *et al*, 2020^97^ | 1 | 0 | 0 | 1 | 0 | 0 | 1 | 1 | 0 | 0 | 4 | moderate |
| Vaillant *et al*, 2019^98^ | 1 | 0 | 0 | 0 | 0 | 0 | 0 | 1 | 0 | 0 | 2 | low |
| Van Horrik *et al*, 2022^99^ | 0 | 0 | 0 | 0 | 0 | 0 | 1 | 0 | 0 | 0 | 1 | low |
| Vazquez *et al*, 2022^100^ | 1 | 0 | 0 | 0 | 0 | 0 | 0 | 0 | 0 | 0 | 1 | low |
| Venugopalan *et al*, 2016^101^ | 1 | 0 | 1 | 1 | 0 | 0 | 1 | 0 | 0 | 0 | 4 | moderate |
| Vernooy *et al*, 2022^102^ | 1 | 0 | 1 | 1 | 0 | 1 | 1 | 0 | 0 | 1 | 6 | moderate |
| Walaszek *et al*, 2017^103^ | 1 | 0 | 0 | 1 | 0 | 0 | 1 | 1 | 0 | 0 | 4 | moderate |
| Weier *et al*, 2017^104^ | 0 | 0 | 0 | 1 | 0 | 1 | 1 | 0 | 0 | 0 | 3 | low |
| Weier *et al*, 2018^105^ | 1 | 0 | 0 | 1 | 0 | 1 | 1 | 0 | 0 | 0 | 4 | moderate |
| Wiese-Possel *et al*, 2023^106^ | 1 | 1 | 0 | 1 | 0 | 0 | 0 | 0 | 0 | 0 | 3 | low |
| Wilcock *et al*, 2016^107^ | 1 | 0 | 1 | 0 | 0 | 1 | 1 | 0 | 0 | 1 | 5 | moderate |
| Wilcox *et al*, 2019^108^ | 1 | 0 | 0 | 1 | 0 | 1 | 1 | 1 | 0 | 1 | 6 | moderate |
| Woodard *et al*, 2023^109^ | 0 | 0 | 0 | 1 | 0 | 1 | 1 | 1 | 0 | 0 | 4 | moderate |
| Xenaki *et al*, 2020^110^ | 1 | 0 | 0 | 1 | 0 | 1 | 0 | 0 | 0 | 0 | 3 | low |
| Zainaghi *et al*, 2023^111^ | 0 | 0 | 0 | 1 | 0 | 1 | 1 | 0 | 0 | 1 | 4 | moderate |
| Zetts *et al*, 2020^112^ | 0 | 0 | 0 | 1 | 0 | 0 | 0 | 0 | 0 | 1 | 2 | low |
| Zgliczynski *et al*, 2022^113^ | 1 | 0 | 0 | 0 | 0 | 1 | 0 | 0 | 0 | 1 | 3 | low |

Q1. Was the study’s target population a close representation of the national population in relation to relevant variables?

Q2. Was the sampling frame a true or close representation of the target population?

Q3. Was some form of random selection used to select the sample, OR was a census undertaken?

Q4. Was the likelihood of nonresponse bias minimal?(Say yes if response rate is higher than 60%; consider other issues such as differences in characteristics of responders and non-responders, if presented)

Q5. Were data collected directly from the subjects (as opposed to a proxy)?

Q6. Was an acceptable case definition used in the study? (In the report, are the knowledge, attitude and practice questions clear?)

Q7. Was the study instrument that measured the parameter of interest shown to have validity and reliability?

Q8. Was the same mode of data collection used for all subjects?

Q9. Was the length of the shortest prevalence period for the parameter of interest appropriate? (PLEASE INCLUDE THE RECALL PERIOD IF STATED. Say yes (0) if survey is measuring KAP without recall period. Say no if the recall period is more than four weeks. For example, if HCPs were asked to report their practice over the past three months then it would be a no (1))

Q10. Were the numerator(s) and denominator(s) for the parameter of interest appropriate? (Yes (0) if they report the actual numbers and not just proportions (and the values are appropriate). Say no (1) if they did not report numbers)

**Reference**

1. Abbas S, Lee K, Pakyz A*, et al.* Knowledge, attitudes, and practices of bedside nursing staff regarding antibiotic stewardship: A cross-sectional study. *American journal of infection control* 2019; **47**: 230-3.

2. Alcalá HE, Maxwell GL, Lindsay B*, et al.* Examining HPV Vaccination Practices and Differences Among Providers in Virginia. *Journal of Cancer Education* 2020; **35**: 159-64.

3. Ashiru-Oredope D, Hopkins S, Vasandani S*, et al.* Healthcare workers' knowledge, attitudes and behaviours with respect to antibiotics, antibiotic use and antibiotic resistance across 30 EU/EEA countries in 2019. *Euro surveillance : bulletin Europeen sur les maladies transmissibles = European communicable disease bulletin* 2021; **26**.

4. Ashiru-Oredope D, Casale E, Harvey E*, et al.* Knowledge and Attitudes about Antibiotics and Antibiotic Resistance of 2404 UK Healthcare Workers. *Antibiotics (Basel, Switzerland)* 2022; **11**.

5. Barchitta M, Sabbatucci M, Furiozzi F*, et al.* Knowledge, attitudes and behaviors on antibiotic use and resistance among healthcare workers in Italy, 2019: investigation by a clustering method. *Antimicrobial resistance and infection control* 2021; **10**: 134.

6. Baudet A, Kichenbr C, Pulcini C*, et al.* Antibiotic use and resistance: a nationwide questionnaire survey among French dentists. *European journal of clinical microbiology & infectious diseases : official publication of the European Society of Clinical Microbiology* 2020; **39**: 1295-303.

7. Belan M, Agrinier N, Gonthier D*, et al.* Antibiotic stewardship in French nursing homes: a 2019 regional survey. *JAC Antimicrob Resist* 2021; **15**.

8. Benedict Kpozehouen E, Arrudsivah B, Tan TC*, et al.* Knowledge, attitudes and practices of health care workers in a cardiology department on influenza vaccination. *Vaccine* 2023; **41**: 2349-56.

9. Beovic B, Dousak M, Pulcini C*, et al.* Young doctors' perspectives on antibiotic use and resistance: a multinational and inter-specialty cross-sectional European Society of Clinical Microbiology and Infectious Diseases (ESCMID) survey. *The Journal of antimicrobial chemotherapy* 2019; **74**: 3611-8.

10. Bianco A, Licata F, Trovato A*, et al.* Antibiotic-Dispensing Practice in Community Pharmacies: Results of a Cross-Sectional Study in Italy. *Antimicrobial agents and chemotherapy* 2021; **65**.

11. Bianco A, Cautela V, Napolitano F*, et al.* Appropriateness of antibiotic prescription for prophylactic purposes among italian dental practitioners: Results from a cross-sectional study. *Antibiotics* 2021; **10**: 547.

12. Bouchoucha SL, Kilpatrick M, Phillips NM*, et al.* Nursing students' awareness and perceptions of nurses' role in antimicrobial stewardship. *Nurse education in practice* 2021; **52**: 103036.

13. Bounou L, Katelani S, Panagiotopoulou K*, et al.* Hand hygiene education of Greek medical and nursing students: A cross-sectional study. *Nurse Education in Practice* 2021; **54**: N.PAG-N.PAG.

14. Briquet C, Khaouch Y, Yombi JC. Perceptions, attitudes, and practices of a Belgian teaching hospital's physicians, pharmacists, and nurses regarding antibiotic use and resistance: survey towards targeted actions for Antimicrobial Stewardship. *Antimicrobial resistance and infection control* 2023; **12**: 19.

15. Buckel WR, Hersh AL, Pavia AT*, et al.* Antimicrobial stewardship knowledge, attitudes, and practices among health care professionals at small community hospitals. *Hospital Pharmacy* 2016; **51**: 149-57.

16. Bunting SR, Miller KW, Chappell R*, et al.* Physician Assistant's Knowledge and Confidence Regarding Prescribing Preexposure Prophylaxis for HIV Prevention. *Sexually Transmitted Diseases* 2020; **47**: 530-4.

17. Carlsson F, Jacobsson G, Lampi E. Antibiotic prescription: Knowledge among physicians and nurses in western Sweden. *Health policy (Amsterdam, Netherlands)* 2023; **130**: 104733.

18. Cataldi JR, O'Leary ST, Beaty BL*, et al.* Measles Experience, Practice, and Knowledge by Pediatricians in the Context of Resurgent US Outbreaks. *Journal of Pediatrics* 2022; **246**: 213-9.e1.

19. Catton T, Umpleby H, Dushianthan A*, et al.* Provision of Microbiology, Infection Services and Antimicrobial Stewardship in Intensive Care: A Survey across the Critical Care Networks in England and Wales. *Antibiotics-Basel* 2023; **12**.

20. Clemence MCS, Henderson A, Greenbury CB*, et al.* A survey of the antibiotic prescribing practices of doctors in an Australian Emergency Department. *Infection, Disease and Health* 2018; **23**: 67-73.

21. Corrente M, Trotta A, Marinaro M*, et al.* Basic knowledge and misconceptions on antibiotic use: a comparative survey between Veterinary College and High School students in Bari (Italy). *Veterinaria italiana* 2021; **57**.

22. Di Gennaro F, Marotta C, Amicone M*, et al.* Italian young doctors' knowledge, attitudes and practices on antibiotic use and resistance: A national cross-sectional survey. *Journal of global antimicrobial resistance* 2020; **23**: 167-73.

23. Evans B, Kosar J, Peermohamed S. Attitudes and Perceptions amongst Critical Care Physicians towards Handshake Antimicrobial Stewardship Rounds. *Cureus Journal of Medical Science* 2019; **11**.

24. Foote A, El-Masri M. Self-perceived hand hygiene practices among undergraduate nursing students. *Journal of Research in Nursing* 2016; **21**: 8-19.

25. Galanis P, Kokkoliou K, Vraka I*, et al.* Healthcare Professionals' Knowledge and Practices towards Hospital Infections in Surgical Clinics. *International Journal of Caring Sciences* 2021; **14**: 1558-67.

26. Golding SE, Higgins HM, Ogden J. Assessing Knowledge, Beliefs, and Behaviors around Antibiotic Usage and Antibiotic Resistance among UK Veterinary Students: A Multi-Site, Cross-Sectional Survey. *Antibiotics* 2022; **11**: 256.

27. Green J, Gardiner SJ, Clarke SL*, et al.* Antimicrobial stewardship practice in New Zealand's rural hospitals. *The New Zealand medical journal* 2018; **131**: 16-26.

28. Hamidi M, Blatz MA. A National Survey of Neonatal Nurses' Knowledge, Beliefs, and Practices of Antibiotic Stewardship. *Advances in neonatal care : official journal of the National Association of Neonatal Nurses* 2023; **23**: E22-E8.

29. Merrill K, Hanson S, Sumner S*, et al.* Antimicrobial stewardship: Staff nurse knowledge and attitudes. *American journal of infection control* 2019; **47**: 1219-24.

30. Hamilton RM, Merrill KC, Luthy KE*, et al.* Knowledge, attitudes, and perceptions of nurse practitioners about antibiotic stewardship. *Journal of the American Association of Nurse Practitioners* 2020; **33**: 909-15.

31. Hammerschmidt J, Manser T. Nurses' knowledge, behaviour and compliance concerning hand hygiene in nursing homes: a cross-sectional mixed-methods study. *BMC Health Services Research* 2019; **19**.

32. Hammoud S, Amer F, Kocsis B. Examining the effect of infection prevention and control awareness among nurses on patient and family education: A cross-sectional study. *Nursing & health sciences* 2022; **24**: 140-51.

33. Hanna LA, Mc Michael C, Hall M. What do future pharmacists know about, and think of, antimicrobial stewardship? *Pharmacy Education* 2019; **19**: 146-54.

34. Harris A, Chandramohan S, Awali RA*, et al.* Physicians' attitude and knowledge regarding antibiotic use and resistance in ambulatory settings. *American Journal of Infection Control* 2019; **47**: 864-8.

35. Hubber J, Person A, Jecha L*, et al.* Knowledge, attitudes, and practices regarding coccidioidomycosis among healthcare providers in four counties in Washington State, 2017. *Medical mycology* 2020; **58**: 730-6.

36. Hurley LP, O'Leary ST, Dooling K*, et al.* Survey of Physician Practices, Attitudes, and Knowledge Regarding Recombinant Zoster Vaccine. *Journal of General Internal Medicine* 2023; **38**: 986-93.

37. Inacio J, Barnes L-M, Jeffs S*, et al.* Master of Pharmacy students' knowledge and awareness of antibiotic use, resistance and stewardship. *Currents in pharmacy teaching & learning* 2017; **9**: 551-9.

38. Jones E, Cope A. Knowledge and attitudes of recently qualified dentists working in Wales towards antimicrobial prescribing and resistance. *European journal of dental education : official journal of the Association for Dental Education in Europe* 2018; **22**: e730-e6.

39. Jones LF, Verlander NQ, Lecky DM*, et al.* Self-reported antimicrobial stewardship practices in primary care using the target antibiotics self-assessment tool. *Antibiotics* 2020; **9**: 253.

40. Kalu IC, Mukhopadhyay S, Dukhovny D*, et al.* Knowledge, Attitudes, and Perceptions about Antibiotic Stewardship Programs among Neonatology Trainees. *American Journal of Perinatology* 2023; **40**: 893-7.

41. Kirk J, Kendall A, Marx JF*, et al.* Point of care hand hygiene-where's the rub? A survey of US and Canadian health care workers' knowledge, attitudes, and practices. *American journal of infection control* 2016; **44**: 1095-101.

42. Kiss CR, Lau JSY, Yeung A*, et al.* Infectious diseases physician attitudes to long-term antibiotic use. *International journal of clinical pharmacy* 2019; **41**: 18-21.

43. Kistler CE, Beeber A, Becker-Dreps S*, et al.* Nursing home nurses' and community-dwelling older adults' reported knowledge, attitudes, and behavior toward antibiotic use. *BMC Nursing* 2017; **16**: 1-7.

44. Kochlamazashvili M, Kamkamidze G, McNutt L-A*, et al.* Knowledge, attitudes and practice survey on blood-borne diseases among dental health care workers in Georgia. *Journal of infection in developing countries* 2018; **12**: 864-70.

45. Kufel WD, Mastro KA, Mogle BT*, et al.* Providers' knowledge and perceptions regarding antibiotic stewardship and antibiotic prescribing in rural primary care clinics. *JACCP Journal of the American College of Clinical Pharmacy* 2020; **3**: 601-8.

46. Lagadinou M, Tsami E, Deligakis A*, et al.* Knowledge and Attitudes of Healthcare Workers towards Antibiotic Use and Antimicrobial Resistance in Two Major Tertiary Hospitals in Western Greece. *Antibiotics (Basel, Switzerland)* 2023; **12**.

47. Lebentrau S, Gilfrich C, Vetterlein MW*, et al.* Impact of the medical specialty on knowledge regarding multidrug-resistant organisms and strategies toward antimicrobial stewardship. *International Urology and Nephrology* 2017; **49**: 1311-8.

48. Lee Y, Bradley N. Antimicrobial Stewardship Practices in a Subset of Community Pharmacies across the United States. *Pharmacy* 2023; **11**: 26.

49. Levin C, Thilly N, Dousak M*, et al.* Perceptions, attitudes, and practices of French junior physicians regarding antibiotic use and resistance. *Medecine et maladies infectieuses* 2019; **49**: 241-9.

50. Li C, Sotomayor-Castillo C, Nahidi S*, et al.* Emergency clinicians' knowledge, preparedness and experiences of managing COVID-19 during the 2020 global pandemic in Australian healthcare settings. *Australasian emergency care* 2021; **24**: 186-96.

51. Llor C, Cordoba G, de Oliveira SM*, et al.* Antibiotic deprescribing: Spanish general practitioners' views on a new strategy to reduce inappropriate use of antibiotics in primary care. *European Journal of General Practice* 2022; **28**: 217-23.

52. Loume A, Gardelis P, Zekeridou A*, et al.* A survey on systemic antibiotic prescription among dentists in Romandy. *Swiss dental journal* 2023; **133**: 800-8.

53. Macintosh JLB, Eden LM, Luthy KE*, et al.* NICU Nurses' Knowledge and Attitudes Regarding 2-Month Immunizations. *Advances in Neonatal Care* 2020; **20**: E111-E7.

54. Marta-Costa A, Mir, a C*, et al.* Survey of the Knowledge and Use of Antibiotics among Medical and Veterinary Health Professionals and Students in Portugal. *International journal of environmental research and public health* 2021; **18**.

55. Mazinska B, Hryniewicz W. Polish physicians' attitudes towards antibiotic prescription and antimicrobial resistance. *Polish Journal of Microbiology* 2017; **66**: 309-19.

56. McCarthy M, Andrews R, Banach DB. Prophylactic antibiotics prior to dental procedures: A cross-disciplinary survey of dentists and medical providers. *American Journal of Infection Control* 2020; **48**: 116-8.

57. McClelland JW, Norris JM, Dominey-Howes D*, et al.* Knowledge and perceptions of Australian postgraduate veterinary students prior to formal education of antimicrobial use and antimicrobial resistance. *One Health* 2022; **14**.

58. Menard C, Fegueux S, Heritage Z*, et al.* Perceptions and attitudes about antibiotic resistance in the general public and general practitioners in France. *Antimicrobial resistance and infection control* 2022; **11**: 124.

59. Mitchell BG, Russo PL, Kiernan M*, et al.* Nurses' and midwives' cleaning knowledge, attitudes and practices: An Australian study. *Infect Dis Health* 2021; **26**: 55-62.

60. Montebello JA, Granick JL, Bollig ER*, et al.* Variation in knowledge, attitude, and practices toward antibiotic use among diplomates of the American Veterinary Dental College: a survey-based study. *Journal of the American Veterinary Medical Association* 2023; **261**: S6-S13.

61. Moriceau G, Gagneux-Brunon A, Gagnaire J*, et al.* Preventing healthcare-associated infections: Residents and attending physicians need better training in advanced isolation precautions. *Medecine et Maladies Infectieuses* 2016; **46**: 14-9.

62. Nelson NP, Allison MA, Lindley MC*, et al.* Physician Knowledge and Attitudes About Hepatitis A and Current Practices Regarding Hepatitis A Vaccination Delivery. *Academic Pediatrics* 2017; **17**: 562-70.

63. Padigos J, Ritchie S, Lim AG. Enhancing nurses' future role in antimicrobial stewardship. *Collegian* 2020; **27**: 487-98.

64. Papini F, Mazzilli S, Paganini D*, et al.* Healthcare Workers Attitudes, Practices and Sources of Information for COVID-19 Vaccination: An Italian National Survey. *International Journal of Environmental Research and Public Health* 2022; **19**: 733.

65. Peres D, Severo M, Ferreira MA. Knowledge, source of information, and perception of Portuguese medical students and junior doctors of infection control precautions. *American journal of infection control* 2016; **44**: 1723-5.

66. Perozziello A, Lescure FX, Truel A*, et al.* Prescribers' experience and opinions on antimicrobial stewardship programmes in hospitals: a French nationwide survey. *The Journal of antimicrobial chemotherapy* 2019; **74**: 2451-8.

67. Pettke A, Jocham S, Wiener A*, et al.* Vaccination against influenza at a European pediatric cancer center: immunization rates and attitudes among staff, patients, and their families. *Supportive Care in Cancer* 2017; **25**: 3815-22.

68. Peytremann A, Senn N, Mueller Y. Infection prevention and control measures in practices of the Swiss sentinel network during seasonal influenza epidemics. *The Journal of hospital infection* 2020; **106**: 786-92.

69. Platace D, Millere I, editors. Motivating factors of infection control in nurse practice. 6th International Interdisciplinary Scientific Conference on Society, Health, Welfare; 2016; Riga.

70. Posada CJ, Boyd LD, Perry KR*, et al.* Knowledge, Attitudes, Practices of Dental Professionals Regarding the Infection Control Guidelines for Dentistry Prior to the COVID-19 Pandemic. *Journal of Dental Hygiene* 2021; **95**: 25-32.

71. Rabano-Blanco A, Dominguez-Martis EM, Mosteiro-Miguens DG*, et al.* Nursing students' knowledge and awareness of antibiotic use, resistance and stewardship: A descriptive cross-sectional study. *Antibiotics* 2019; **8**: 203.

72. Ricco M, Ferraro P, Camisa V*, et al.* When a Neglected Tropical Disease Goes Global: Knowledge, Attitudes and Practices of Italian Physicians towards Monkeypox, Preliminary Results. *Tropical Medicine and Infectious Disease* 2022; **7**: 135.

73. Ricco M, Zaniboni A, Satta E*, et al.* West Nile Virus Infection: A Cross-Sectional Study on Italian Medical Professionals during Summer Season 2022. *Tropical Medicine and Infectious Disease* 2022; **7**: 404.

74. Ricco M, Ferraro P, Peruzzi S*, et al.* Respiratory Syncytial Virus: Knowledge, Attitudes and Beliefs of General Practitioners from North-Eastern Italy (2021). *Pediatric Reports* 2022; **14**: 147-65.

75. Ricco M, Vezzosi L, Gualerzi G*, et al.* Knowledge, attitudes, beliefs and practices of obstetrics-gynecologists on seasonal influenza and pertussis immunizations in pregnant women: Preliminary results from North-Western Italy. *Minerva Ginecologica* 2019; **71**: 288-97.

76. Rodrigues AT, Nunes JCF, Estrela M*, et al.* Comparing hospital and primary care physicians' attitudes and knowledge regarding antibiotic prescribing: A survey within the centre region of Portugal. *Antibiotics* 2021; **10**: 629.

77. Saha SK, Kong DCM, Thursky K*, et al.* A nationwide survey of australian general practitioners on antimicrobial stewardship: Awareness, uptake, collaboration with pharmacists and improvement strategies. *Antibiotics* 2020; **9**: 1-13.

78. Saha SK, Kong DCM, Thursky K*, et al.* Antimicrobial stewardship by Australian community pharmacists: Uptake, collaboration, challenges, and needs. *Journal of the American Pharmacists Association* 2021; **61**: 158-68.e7.

79. Sahai V, Eden K, Glustein S*, et al.* Hand hygiene knowledge, attitudes and self-reported behaviour in family medicine residents. *Canadian Journal of Infection Control / Revue Canadienne de Prévention des Infections* 2016; **31**: 11-7.

80. Sakeena MHF, Bennett AA, McLachlan AJ. Investigating knowledge regarding antibiotics and antimicrobial resistance among pharmacy students in Australian universities. *Journal of Pharmacy Practice and Research* 2021; **51**: 54-61.

81. Salm F, Schneider S, Schmucker K*, et al.* Antibiotic prescribing behavior among general practitioners - a questionnaire-based study in Germany. *BMC infectious diseases* 2018; **18**: 208.

82. Salsgiver E, Bernstein D, Simon MS*, et al.* Knowledge, Attitudes, and Practices Regarding Antimicrobial Use and Stewardship Among Prescribers at Acute-Care Hospitals. *Infection control and hospital epidemiology* 2018; **39**: 316-22.

83. Saveanu CI, Darabaneanu G, Bobu LI*, et al.* A Cross-Sectional Questionnaire-Based Survey on Blood-Borne Infection Control among Romanian Dental Students. *Medicina-Lithuania* 2022; **58**.

84. Schneider S, Salm F, Vincze S*, et al.* Perceptions and attitudes regarding antibiotic resistance in Germany: a cross-sectoral survey amongst physicians, veterinarians, farmers and the general public. *The Journal of antimicrobial chemotherapy* 2018; **73**: 1984-8.

85. Schneider F, Schulz CM, May M*, et al.* The association of the anesthesiologist's academic and educational status with self-confidence, self-rated knowledge and objective knowledge in rational antibiotic application. *BMC Research Notes* 2020; **13**: 161.

86. Shukla PJ, Behnam-Terneus M, Cunill-De Sautu B*, et al.* Antibiotic use by pediatric residents: Identifying opportunities and strategies for antimicrobial stewardship. *Hospital Pediatrics* 2017; **7**: 553-8.

87. Simoes A, Alves D, Gregorio J*, et al.* Fighting antibiotic resistance in Portuguese hospitals: Understanding antibiotic prescription behaviours to better design antibiotic stewardship programmes. *Journal of global antimicrobial resistance* 2018; **13**: 226-30.

88. Smith CR, Pogany L, Foley S*, et al.* Canadian physicians' knowledge and counseling practices related to antibiotic use and antimicrobial resistance: Two-cycle national survey. *Canadian family physician Medecin de famille canadien* 2017; **63**: e526-e35.

89. Sobierajski T, Mazinska B, Wanke-Rytt M*, et al.* Knowledge-Based Attitudes of Medical Students in Antibiotic Therapy and Antibiotic Resistance. A Cross-Sectional Study. *International journal of environmental research and public health* 2021; **18**.

90. Spernovasilis N, Ierodiakonou D, Milioni A*, et al.* Assessing the knowledge, attitudes and perceptions of junior doctors on antimicrobial use and antimicrobial resistance in Greece. *Journal of Global Antimicrobial Resistance* 2020; **21**: 296-302.

91. Steinberg M, Dresser LD, Daneman N*, et al.* A National Survey of Critical Care Physicians' Knowledge, Attitudes, and Perceptions of Antimicrobial Stewardship Programs. *Journal of intensive care medicine* 2016; **31**: 61-5.

92. Struzycka I, Mazinska B, Bachanek T*, et al.* Knowledge of antibiotics and antimicrobial resistance amongst final year dental students of Polish medical schools-A cross-sectional study. *European journal of dental education : official journal of the Association for Dental Education in Europe* 2019; **23**: 295-303.

93. Szumska E, Czajkowski P, Zablocki M*, et al.* The Association between Hand Disinfection Techniques and Their Barriers, as Well as the "Bare below the Elbows" Concept, among Healthcare Professionals-A Study Based on a Polish Population. *International Journal of Environmental Research and Public Health* 2022; **19**: 11781.

94. Tomczyk S, Whitten T, Holzbauer SM*, et al.* Combating antibiotic resistance: a survey on the antibiotic-prescribing habits of dentists. *General Dentistry* 2018; **66**: 61-8.

95. Torres E, Richman A, Wright W*, et al.* Assessing Dental Students' HPV Health Literacy and Intention to Engage in HPV-Related Oropharyngeal Cancer Prevention. *Journal of cancer education : the official journal of the American Association for Cancer Education* 2022; **37**: 950-6.

96. Trautner BW, Greene MT, Krein SL*, et al.* Infection Prevention and Antimicrobial Stewardship Knowledge for Selected Infections Among Nursing Home Personnel. *Infection control and hospital epidemiology* 2017; **38**: 83-8.

97. Trucchi C, Restivo V, Amicizia D*, et al.* Italian health care workers' knowledge, attitudes, and practices regarding human papillomavirus infection and prevention. *International Journal of Environmental Research and Public Health* 2020; **17**: 1-12.

98. Vaillant L, Birg G, Esposito-Farese M*, et al.* Awareness among French healthcare workers of the transmission of multidrug resistant organisms: a large cross-sectional survey. *Antimicrobial resistance and infection control* 2019; **8**: 173.

99. van Horrik TMZXK, Laan BJ, Platteel TN*, et al.* Guideline Adherence of Asymptomatic Bacteriuria Could Be Improved among General Practitioners in The Netherlands: A Survey Study. *Antibiotics* 2022; **11**: 75.

100. Vazquez Guillamet MC, Burnham JP, Perez M*, et al.* Antimicrobial stewardship for sepsis in the intensive care unit: Survey of critical care and infectious diseases physicians. *Infection control and hospital epidemiology* 2022; **43**: 1368-74.

101. Venugopalan V, Trustman N, Manning N*, et al.* Administration of a survey to evaluate the attitudes of house staff physicians towards antimicrobial resistance and the antimicrobial stewardship programme at a community teaching hospital. *Journal of global antimicrobial resistance* 2016; **4**: 21-7.

102. Vernooy CP, Ang A, Emond Y*, et al.* Assessment of antimicrobial prescribing practice, knowledge, and culture in three teaching hospitals. *JAMMI* 2022; **7**: 317-22.

103. Walaszek M, Kolpa M, Wolak Z*, et al.* Poor hand hygiene procedure compliance among Polish medical students and physicians-the result of an ineffective education basis or the impact of organizational culture? *International Journal of Environmental Research and Public Health* 2017; **14**: 1026.

104. Weier N, Thursky K, Zaidi STR. Antimicrobial knowledge and confidence amongst final year medical students in Australia. *PLoS One* 2017; **12**: e0182460.

105. Weier N, Tebano G, Thilly N*, et al.* Pharmacist participation in antimicrobial stewardship in Australian and French hospitals: a cross-sectional nationwide survey. *Journal of Antimicrobial Chemotherapy* 2018; **73**: 804-13.

106. Wiese-Posselt M, Lam T-T, Schroder C*, et al.* Appropriate antibiotic use and antimicrobial resistance: knowledge, attitudes and behaviour of medical students and their needs and preferences for learning. *Antimicrobial resistance and infection control* 2023; **12**: 48.

107. Wilcock M, Wisner K, Powell N. GPs' perceptions of AMR and antimicrobial stewardship. *Prescriber* 2016; **27**: 44-7.

108. Wilcox CR, Calvert A, Metz J*, et al.* Attitudes of Pregnant Women and Healthcare Professionals Toward Clinical Trials and Routine Implementation of Antenatal Vaccination Against Respiratory Syncytial Virus: A Multicenter Questionnaire Study. *Pediatric Infectious Disease Journal* 2019; **38**: 944-51.

109. Woodard L, Gilbert L, King B*, et al.* Examining Black and Hispanic physicians and other healthcare providers' attitudes toward the COVID-19 vaccine. *Journal of the National Medical Association* 2023; **115**: 53-65.

110. Xenaki D, Plotas P, Michail G*, et al.* Knowledge, behaviours and attitudes for human papillomavirus (HPV) prevention among educators and health professionals in Greece. *European Review for Medical and Pharmacological Sciences* 2020; **24**: 7745-52.

111. Zainaghi I, Cilluffo S, Lusignani M. Knowledge, attitudes, and practices related to antibiotic resistance among physicians and nurses in Italian intensive care: A multicenter cross-sectional survey. *Journal of Global Antimicrobial Resistance* 2023; **36**.

112. Zetts RM, Garcia AM, Doctor JN*, et al.* Primary care physicians' attitudes and perceptions towards antibiotic resistance and antibiotic stewardship: A national survey. *Open Forum Infectious Diseases* 2020; **7**.

113. Zgliczynski WS, Bartosinski J, Rostkowska OM. Knowledge and Practice of Antibiotic Management and Prudent Prescribing among Polish Medical Doctors. *International journal of environmental research and public health* 2022; **19**.
